# Anaemia is associated with severe RBC dysfunction and a reduced circulating NO pool: vascular and cardiac eNOS are crucial for the adaptation to anaemia

**DOI:** 10.1007/s00395-020-0799-x

**Published:** 2020-06-12

**Authors:** Patricia Wischmann, Viktoria Kuhn, Tatsiana Suvorava, Johanna M. Muessig, Jens W. Fischer, Brant E. Isakson, Sebastian M. Haberkorn, Ulrich Flögel, Jürgen Schrader, Christian Jung, Miriam M. Cortese-Krott, Gerd Heusch, Malte Kelm

**Affiliations:** 1 Department of Cardiology, Pulmonary Diseases, and Vascular Medicine, Medical Faculty, CARID Cardiovascular Research Institute of Duesseldorf, Heinrich Heine University of Duesseldorf, Moorenstr. 5, 40225 Düsseldorf, Germany; 2 Division of Cardiology, Pulmonary Diseases and Vascular Medicine, University Hospital of Duesseldorf, Düsseldorf, Germany; 3 Cardiovascular Research Laboratory, Medical Faculty, Heinrich-Heine University, Düsseldorf, Germany; 4 Department of Pharmacology and Clinical Pharmacology, Heinrich-Heine University, Düsseldorf, Germany; 5 Department of Molecular Physiology and Biological Physics, Robert M. Berne Cardiovascular Research Centre, University of Virginia School of Medicine, Charlottesville, VA USA; 6 Department of Molecular Cardiology, Heinrich Heine University, Düsseldorf, Germany; 7 Institute for Pathophysiology, West German Heart and Vascular Centre, University of Essen Medical School, Essen, Germany

**Keywords:** Acute myocardial infarction, Anaemia, Endothelial nitric oxide synthase, Mortality, Red blood cell function

## Abstract

Anaemia is frequently present in patients with acute myocardial infarction (AMI) and contributes to an adverse prognosis. We hypothesised that, besides reduced oxygen carrying capacity, anaemia is associated with (1) red blood cell (RBC) dysfunction and a reduced circulating nitric oxide (NO) pool, (2) compensatory enhancement of vascular and cardiac endothelial nitric oxide synthase (eNOS) activity, and (3) contribution of both, RBC dysfunction and reduced circulatory NO pool to left ventricular (LV) dysfunction and fatal outcome in AMI. In mouse models of subacute and chronic anaemia from repeated mild blood loss the circulating NO pool, RBC, cardiac and vascular function were analysed at baseline and in reperfused AMI. In anaemia, RBC function resulted in profound changes in membrane properties, enhanced turnover, haemolysis, dysregulation of intra-erythrocytotic redox state, and RBC-eNOS. RBC from anaemic mice and from anaemic patients with acute coronary syndrome impaired the recovery of contractile function of isolated mouse hearts following ischaemia/reperfusion. In anaemia, the circulating NO pool was reduced. The cardiac and vascular adaptation to anaemia was characterised by increased arterial eNOS expression and activity and an eNOS-dependent increase of end-diastolic left ventricular volume. Endothelial dysfunction induced through genetic or pharmacologic reduction of eNOS-activity abrogated the anaemia-induced cardio-circulatory compensation. Superimposed AMI was associated with decreased survival. In summary, moderate blood loss anaemia is associated with severe RBC dysfunction and reduced circulating NO pool. Vascular and cardiac eNOS are crucial for the cardio-circulatory adaptation to anaemia. RBC dysfunction together with eNOS dysfunction may contribute to adverse outcomes in AMI.

**Electronic supplementary material:**

The online version of this article (10.1007/s00395-020-0799-x) contains supplementary material, which is available to authorized users.

## Introduction

Patients with acute coronary syndromes (ACS) often have anaemia, which contributes to an adverse prognosis [2, 34]. About 30–40% of patients with acute myocardial infarction (AMI) have evident anaemia on admission [2] or develop hospital-acquired anaemia [35]. Severe complications impair the outcome of anaemic patients with AMI, such as thromboembolic events, augmented bleeding [12, 28, 42], hypertension and arrhythmias [5]. Blood transfusion or erythropoiesis stimulating agents have failed to improve outcome in AMI patients with anaemia or were even harmful [2, 3]. This failure has raised the idea that not only changes in the level of oxygen carrying haemoglobin, but also alterations of other red blood cell (RBC) functions interact with the cardiac and circulatory compensation of anaemia and thus worsen the outcome of AMI.

RBC transport nitric oxide (NO), synthesize NO through nitrite reduction under hypoxia, and form NO via an active endothelial nitric oxide synthase (eNOS) during normoxia which [9, 24] contributes to the circulating NO pool [33, 43]. Depletion of circulating NO increases blood pressure, the severity of AMI and left ventricular (LV) remodelling after myocardial infarction [20, 26, 43]. RBC eNOS becomes dysfunctional with diabetes mellitus [45, 47], arterial hypertension, and kidney disease [15]. The eNOS is located within the RBC membrane and affects vascular function acutely and chronically [24, 39]. However, the role of RBC eNOS in anaemia is not known so far. RBC dysfunction in anaemia may impair cardiac and vascular functions independently from and in addition to impaired oxygen delivery.

An upregulation of vascular eNOS may improve flow-mediated dilation, decrease systemic vascular resistance and thus facilitate oxygen delivery to peripheral tissue. Increased cardiac NO bioavailability attenuates left ventricular dysfunction and adverse remodelling post AMI [31, 32]. Thus, it is tempting to speculate, that increases in vascular and cardiac NO bioavailability may contribute to the compensation of anaemia. In AMI, this may be of particular significance, as acute and chronic endothelial dysfunction frequently coincide with major risk factors such as hypertension, hyperlipidaemia, and diabetes mellitus, which might then prevent such beneficial compensation of anaemia and thus impair prognosis in ischaemic heart disease and AMI [11].

Therefore, our objectives were to provide evidence that with anaemia (1) there is severe RBC dysfunction with reduced NO bioavailability and impaired cardiac function, (2) there is an increased vascular and cardiac eNOS activation which mediates the adaptation to anaemia, and (3) there is a contribution of both, RBC dysfunction and reduced NO bioavailability to LV dysfunction and fatal outcome in AMI. To mimic a clinically relevant type of moderate anaemia, we established mouse models of subacute and chronic anaemia from repeated mild blood loss, to investigate the underlying mechanisms of circulatory and cardiac compensation of anaemia in AMI. We further investigated the cardioprotective capabilities of RBC from ACS patients with and without anaemia in an ex vivo mouse heart model of ischaemia/reperfusion.

## Methods

### Induction of subacute and chronic anaemia

Moderate anaemia was induced in healthy, 10- to 12-week-old male C57BL/6J (Janvier Labs, Saint-Berthevin Cedex; France) (wild type, WT) mice by mild blood withdrawal (< 20 g/L Hb/day) on 3 consecutive days. The amount of daily blood loss per mouse was adjusted to < 15% of total blood volume and replaced by saline administration. In subacute anaemia, imaging, measurements of hemodynamics and body weight were performed at baseline, on day 3 immediately before induction of AMI, and on day 4 (24 h after AMI). In addition, a protocol for chronic anaemia was established expanding that for subacute anaemia to 6 weeks with repetitive mild blood withdrawal (by < 20 g/L) every 3 days to maintain the target level of haemoglobin 90 g/L. Mice underwent once a week echocardiographic analysis of LV function before blood was collected for further analysis. For the analysis of recovery studies echocardiography and blood analysis were performed in anaemic mice on day 1, 2, 3, 5 and 7 after subacute anaemia had been induced. The techniques, including the facial vein phlebotomy and the retrobulbar sinus puncture, were described previously [38]. Blood samples were analysed no later than 12 h after collection using a scil Vet ABC ™Haematology Analyzer following the manufacturer’s instructions to quantify parameters of blood count. In this analysis, blood withdrawal was limited to no more than 40 µL per day to avoid a further decrease of red blood cells (RBC).

### Analysis of blood volume

To exclude significant changes in circulating blood volume and preload a separate group of anaemic mice underwent blood withdrawal for 3 consecutive days with and without isotonic volume replacement (s.c. and i.p.) by saline each day. These mice underwent echocardiography, invasive catheterization and determination of total blood volume by Evans blue dye at day 0 and 3 [4, 17]. Both mouse groups at day 3 were subjected to an injection of 50 µL of a 0.4% solution (0.4 g/100 mL) of Evans blue in sterile NaCl 0.9% (which corresponds to 0.2 mg Evans blue) intravenous (i.v). After 5 minutes (min), the final heart puncture was performed and at least 500 µL blood were collected. 50 µL were used to measure the haematocrit (Hct), the rest was centrifuged at 3000*g* for 10 min to obtain plasma and kept on ice. The Evans blue plasma concentration was assessed at 610 nm using a spectrophotometer (FLUOstar Omega^®^, S/N: 415–1204). For quantification, standards of plasma dilutions (1:5–1:200) were used. Total blood volume was calculated: *V*_total_ = (*V*_plasma_/(100 − Hct)) × Hct + *V*_plasma_. For normalization, blood volume was divided by body weight. In both groups, blood volume replacement did not affect blood volume (anaemia + isotonic saline 1.25 mL vs. anaemia – isotonic saline 1.40 mL).

### Human subjects

Blood samples were collected from 14 acute coronary syndrome (ACS) patients without anaemia and 15 ACS patients with anaemia, respectively. All patients with ACS were recruited from the department of Cardiology, pulmonology and angiology in Düsseldorf, University Hospital. The diagnosis of anaemia was according to current WHO (World Health Organization) guidelines. ACS was defined by ST-Segment Elevation Myocardial Infarction (STEMI) or non-STEMI according to the appearance of the electrocardiogram. Blood was taken within 24 h after STEMI symptom onset and within 72 h in case of non-STEMI.

### RBC deformability

Blood was collected in heparinized tubes from anaemic and sham mice at day 3. RBC deformability was determined by ektacytometry in a laser-assisted optical rotational cell analyser (LORCA, University of Amsterdam, Netherlands). Whole blood was centrifuged for 3 min at 3,000 g. Plasma was discarded. 30 µL of washed RBC were diluted 1:2 with isotonic saline 0.9% and stored at 4 °C for no longer than 20 min. The samples were then further diluted in 5 mL pre-warmed polyvinylpyrrolidone (PVP) solution (ready-to-use as provided by R&R Mechatronics International B.V., Zwaag, Netherlands), gently stirred and measured according to the user’s manual Version 2.1 with the laser-assisted optical rotational cell analyser. RBC deformability was expressed by the elongation index (EI), which was calculated from the elliptical RBC diffraction pattern as EI = (*L* − *W*)/(*L* − *W*), where *L* and *W* are the length and width of the diffraction pattern, respectively, as described previously [23].

### RBC redox state

For the determination of reduced and total glutathione (GSH), centrifuged and washed RBC and plasma of anaemic and sham mice were homogenised in ice-cold 0.01 M hydrochloric acid (HCl) and sonicated for 30 s at 4 °C. After centrifugation at 14,000*g* for 10 min at 4 °C, the supernatant was mixed with 5% sulfosalicylic acid (SSA) (2.5% final concentration) to precipitate the proteins, and centrifuged again as described above. The clear supernatant was further processed and used for GSH measurements using a commercial kit (GSH DetectX Fluorescent Detection Kit Arbour Assays, Ann Arbour, MI, USA) following the manufacturer’s instructions. Oxidised glutathione (GSSG) was calculated as (total GSH − free GSH)/2. The ratio of free GSH/GSSG was calculated.

### RBC turnover

To measure the amount of CD71+ and of phosphatidylserine positive (P+) RBC, arterial blood was collected in heparinized tubes and processed within 2 h. 30 µL blood was diluted with 15 mL ice-cold phosphate buffered saline (PBS). RBC suspensions were stained with 5 µL CD71-Allophycocyanin (APC) (#130-091-727, Milteny Biotech) and Annexin-V-Phycoerythrin (PE) (#556422, BD Bioscience) for 30 min at 4 °C in the dark. All tubes were centrifuged (300*g*, 10 min, 4 °C), and supernatants were removed. Pellets were resuspended in 1000 µL PBS and analysed via flow cytometry (BD FACSVerse™ flow cytometer, BD Biosciences, San Jose, California, USA). Flow cytometric data were collected using the BD FACSuite, analysed using FlowJo (TreeStar), and calculated as described above. The number of CD71+ or PS+ was calculated as percentage of total gated RBC. Median fluorescence intensity (MFI) was calculated from the histogram (distribution) plots of the green fluorescence signals [extinction (Ex) 488 nm, emission (Em) 530 ± 30 nm] detected within the cell-specific gates. MFIs of untreated samples served as autofluorescence controls [10].

### RBC integrity

For measurement of cell-free plasma Hb and lactate dehydrogenase (LDH), venous blood was taken with heparinized glass pipettes under mild anaesthesia (1.5% isoflurane) by retrobulbar sinus puncture. LDH levels were analysed using ELISA techniques by LDH Assay Kit from (abcam^®^, Cambridge, UK), and cell-free plasma Hb was measured using a DetectX^®^ Haemoglobin Colorimetric Detection Kit from (Arbour Assays, Ann Arbour, MI, USA), each following the manufacturer’s instructions. For measurement of circulating haptoglobin, erythropoietin, and iron, arterial blood obtained by heart puncture was collected in heparinized tubes on day 3 from anaemic and sham mice and centrifuged at 3000*g* for 5 min at 4 °C. Plasma haptoglobin was assessed by the Haptoglobin Mouse ELISA Kit purchased from (abcam^®^, Cambridge, UK). Erythropoietin was analysed in plasma using the Mouse Erythropoietin ELISA Kit purchased from (MyBioSource^®^, San Diego, CA, USA). For analysis of iron, arterial blood was obtained by heart puncture, collected in heparinized syringes and transferred directly into serum collection tubes. After 30 min of incubation at room temperature, tubes were centrifuged at 1000*g* for 15 min in a 4 °C cold microcentrifuge, and serum was immediately processed or frozen and kept at − 80 °C for no longer than 1 month until analysis. Iron levels were analysed using colorimetric measurements with Iron Assay kit purchased from (abcam^®^, Cambridge, UK). Haemolytic samples were excluded from further analysis.

### Oxygen transport in the systemic circulation

Blood samples of about 100 µL each were withdrawn with a heparinized syringe (B. Braun Omnica^®^ F syringe; 1 mL; 30G; 0.3 × 12 mm) after cardiac puncture from the left ventricle and subsequently from the right ventricle to measure oxygenation, lactate, glucose and electrolytes in arterial and central venous blood. Blood gas analysis was performed no later than 5 min after blood withdrawal with the ABL800 FLEX analyser (Radiometer Medical ApS, Brønshøj, Denmark) following the manufacturer’s instructions. Arterial and venous oxygen content was calculated as follows: CaO_2_ = SaO_2_ × Hb (g/dL) × 1.34 (mL/g) + PaO_2_ (mmHg) × 0.0031 (1/mmHg × mL/dL) for arterial and CvO_2_ = SvO_2_ × Hb (g/dL) × 1.34 (mL/g) + PvO_2_ (mmHg) × 0.0031 (1/mmHg × mL/dL) for central venous blood. Hb = haemoglobin; PaO_2_ = arterial partial oxygen pressure; PvO_2_ = venous partial oxygen pressure; SaO_2_ = arterial oxygen saturation; SvO_2_ venous oxygen saturation. Arteriovenous O_2_ difference (avDO_2_) was calculated as avDO_2_ = CaO_2_ − CvO_2_. These parameters were used to calculate O_2_ consumption (VO_2_) as VO_2_ (O_2_ uptake per min) = CO (mL/min) × avDO_2_, and O_2_ delivery (DO_2_) as DO_2_ (O_2_ delivery per min) = CO (mL/min) × CaO_2_.

### Analysis of systemic haemodynamics

Heart rate (HR) and mean arterial blood pressure (MAP) were measured through a 1.4 F Millar pressure-conductance catheter (SPR-839, Millar Instruments, Houston, TX, USA) placed into the right carotid artery [40]. Pressure was recorded by a Millar Box and analysed with LabChart 7 (AD Instruments, Oxford, UK). Systemic vascular resistance (SVR) was calculated as SVR ≅ MAP (mean arterial pressure) / CO (cardiac output). To assess left ventricular end diastolic developed pressure (LVEDP), maximum rate of pressure increase (dP/dtmax) and maximum rate of pressure decrease (dP/dtmin) the Millar catheter was advanced into the left ventricle (LV). Pressure–volume loops were generated using iox software v.2.10.0 (EMKA technologies, Paris, France). These loops were not calibrated for baseline differences in volume parameters since absolute LV volume was more precisely measured by CMRI and echocardiography.

### Analysis of endothelium-dependent vascular function

Vascular function was assessed with a Vevo 2100 high-resolution ultrasound scanner using a 30–70 MHz linear transducer (Visual Sonics Inc., Toronto, Canada). Mice were kept under 1.5–2% isoflurane anaesthesia at a heart rate of 400–500 bpm, breathing rate of about 100 breaths per minute, and 37 °C body temperature. The transducer was placed using a stereotactic holder and adjusted manually to visualise the arteria iliaca externa [16]. A vascular occluder (8 mm diameter, Harvard Apparatus, Harvard, Boston, MA, USA) was placed around the lower limb. Baseline images of the vessel were first recorded, the cuff (positioned above the knee) was then inflated to 200 mmHg, and pressure was kept constant for 5 min (Druckkalibriergerät KAL 84, Halstrup Walcher, Kirchzarten, Germany); then the cuff was deflated to allow reperfusion with enhanced blood flow causing shear stress and consequent flow-mediated dilation (FMD). The upstream diameter of the vessel was determined every 30 s both during inflation and deflation of the cuff. Changes in vessel diameter were quantified as percent of baseline (%) = [diameter (max)/diameter (baseline)] × 100. Measurements were also performed in response to the nitric oxide donor glycerol trinitrate (GTN) 1 and 2 min after its infusion i.p. (12 mg/kg BW). In addition, FMD was analysed after treatment with the nitric oxide synthase (NOS) inhibitor L-NAME [1 mg/mL drinking water (see above)] for 4 days.

### Protocols to assess effects of eNOS in anaemia

To assess the contribution of endothelial nitric oxide synthase (eNOS) to the anaemia-induced circulatory and cardiac compensation at baseline and during AMI, protocols with acute infusion of 2-ethyl-2-thiopseudourea (ETU, Sigma-Aldrich, Steinheim, Germany) via intraperitoneally injection (i.p.: 0.245 µg/µL/min in saline 0.9%) during coronary ligation (left anterior descending coronary artery, LAD) and the first 15 min of reperfusion [26], treatment with *N*(ω)-nitro-l-arginine methyl ester (L-NAME, Sigma-Aldrich, Steinheim, Germany) (1 mg/mL) orally (p.o.) in drinking water 1 day prior to the first puncture for totally 4 days prior to AMI, and in eNOS deficient (eNOS^−/−^) mice with induction of anaemia by blood withdrawal on 3 consecutive days and 2:1 replacement with saline 0.9%, as described for WT mice were performed [19].

### Analysis of NO metabolites

For the analysis of nitric oxide (NO) metabolites, blood and tissue were collected as described previously [8]. At the beginning of the experiment, mice were anaesthetised with isoflurane (2.0%) and blood was taken by heart punction and collected in a heparinized syringe. Afterwards the blood was transferred directly into tubes containing *N*-ethylmaleimide (NEM)/EDTA (10:1 v/v), dissolved in phosphate-buffered solution (PBS) at pH 7.4 (final concentrations: 10 mM NEM, 2 mM EDTA), and centrifuged immediately for 3 min at 3000*g*. Tissue was removed after 1 min of perfusion with ice cold 10 mM NEM/2 mM EDTA in PBS pH 7.4, blotted dry on filter paper, weighed, snap frozen in liquid nitrogen, and kept at − 80 °C until later analysis. Nitrosated (*S*-nitroso and *N*-nitroso) products (RXNO) and NO haem were quantified by gas phase chemiluminescence as described [19]. The analysis of nitrite/nitrate was done in deproteinized NEM-treated samples with ice-cold methanol (1:1 v/v), cleared by centrifugation and subjected to analysis a gas phase chemiluminescence-based technique as well as a high pressure liquid chromatography (HPLC) using a dedicated nitrite/nitrate analyser (ENO20, Eicom) as described [8, 33]. Data are given for the respective volume of plasma and RBC in 1 mL blood volume normalised to haematocrit in mice with comparable body weight.

### Analysis of eNOS expression in aorta and hearts

The Western blot analysis of eNOS in the murine heart and aorta was carried out according to the protocol [26]. The lysis of organs was done with RIPA buffer (1% NP40, 0.5% sodium deoxycholate, and 0.1% SDS in PBS pH 7.4), containing a cocktail of protease and phosphatase inhibitors (Pierce, New Haven, USA). Therefore, samples were homogenised at 4 °C using Tissue Ruptor (Qiagen, Hilden, Germany), sonicated for 3 min at 4 °C, and centrifuged at 4000*g* for 10 min at 4 °C. Before loading the gels, total protein concentration of the supernatant was determined by Lowry assay. The calculated amount of protein from the samples was then loaded in 7% NuPAGE Tris–Acetate precast gels (Invitrogen, Waltham, USA) and transferred onto PVDF membrane Hybond P (Amersham Biosciences, Munich, Germany). The membranes were blocked for 2 h with 5% BSA (Bovine serum albumin) (Bio-Rad, Hercules, California,USA) in T-TBS (10 mM Tris, 100 mM NaCl, 0.1% Tween) at room temperature and finally incubated overnight at 4 °C with a mouse anti-eNOS (1:500, custom made from number 624086 anti-eNOS/NOS type III antibody, stock: 1 mg/mL in PBS pH 7.4, BD Bioscience, Erembodegem, Belgium) or polyclonal rabbit anti-actin (1:1000, product number A2066 Sigma) or mouse monoclonal anti-GAPDH (1:5000, Sigma Aldrich, St Louis MO, USA) in T-TBS. The membranes were then incubated for an hour at room temperature under small rotation before being washed for 1 h in T-TBS. The last step, before detection of the bands, contains the incubation of the membranes with HRP (Horseradish peroxidase)-conjugated goat anti-mouse or anti-rabbit secondary antibodies (1:5000; BD Biosciences). The detection was done using Amersham ECL Select Western Blotting Detection Reagent (number RPN2235, GE Healthcare) and Image Quant (GE Healthcare). Densitometry was carried out using Image Studio Lite software (LI-COR Biotechnology, Lincoln, NE, USA). Detection and quantification of the bands were compared within the linear range of the respective method of analysis.

### Induction of AMI

10–12 weeks old, male C57Bl/6J (wild type, WT) mice were initially anaesthetised with isoflurane, intubated and ventilated at a tidal volume of 0.2–0.25 mL and a respiratory rate of 140 breaths per minute with isoflurane (1.5%) and 21% O_2_ with a rodent ventilator. After a left lateral thoracotomy between the third and fourth rib, the pericardium was dissected, and a 7–0 surgical suture was cautiously passed underneath the LAD artery at a position 1 mm from the tip of the left auricle. AMI was produced by suture occlusion, and a short silicone role was placed into the suture loop to facilitate its reopening. The correct position of the suture was confirmed by blanching of the apex and characteristic changes in the ECG (ST-segment elevation). After 45 min with continuously controlled body temperature (37 °C), the ligation was removed and the myocardium reperfused for 24 h. Animals received buprenorphine (0.5 mg/kg BW) s.c. every 8 h until euthanasia.

### Analysis of myocardial area at risk and infarct size

Mice were anaesthetised by i.p. injection of 100 mg/kg ketamine (Ketanest^®^) and 10 mg/kg xylazine (Rompun^®^) and anticoagulated with heparin (1000 IU i.p.). Hearts were rapidly excised and transferred to cold, oxygenated Krebs–Henseleit buffer. Evans blue dye (1 mL of a 1% solution) was injected into the aorta and coronary arteries for delineation of the ischaemic area at risk (AAR) from the non-ischaemic zone. The tissue was wrapped in a clear food wrap and stored for 1 h in a − 20 °C freezer. The heart was then serially sectioned perpendicularly to the long axis in 1 mm slices, and each slice was weighed. The sections were incubated in 1% TTC for 5 min at 37 °C for demarcation of the viable and non-viable myocardium within the risk zone. The areas of infarction, AAR, and non-ischaemic LV were assessed with computer-assisted planimetry by an observer blinded to sample identity. Final infarct sizes are expressed as percent of AAR. For all biochemical analyses, hearts were perfused free of blood, excised, snap-frozen in liquid nitrogen and stored at -80 °C until further analysis [7]. Troponin T levels were measured using a high sensitivity Troponin T (cTnT hs) Assay (Roche Diagnostics, Basel, Switzerland).

### Echocardiography to assess LV function

Echocardiography: cardiac images were acquired [20] using a Vevo 2100 high-resolution ultrasound scanner (18–38 MHz linear array micro scan transducer; Visual Sonics Inc., Toronto, Canada) and the manufacturer’s analysis software on day 3 in anaemic and sham mice and 24 h after reperfused AMI in both groups. In addition, echocardiography was performed in a group each of anaemic and non-anaemic mice before AMI, 4 and 24 h, 4 and 7 days after reperfused AMI. Left ventricular (LV) volumes, stroke volume (SV), cardiac output (CO) and ejection fraction (EF) were calculated using B-mode for identification of maximal and minimal cross-sectional area [1, 36]. To assess regional function, indexes of LV reshaping, and infarct size were analysed by cardiac magnetic resonance imaging.

### Cardiac magnetic resonance imaging

Cardiac magnetic resonance imaging (cMRI) data were recorded at a Bruker AVANCEIII 9.4 T wide bore NMR spectrometer (Bruker, Rheinstetten, Germany) at 400.13 MHz operated by ParaVision 5.1. Images were acquired using the Bruker microimaging unit Micro 2.5 with actively shielded gradient sets (1.5 T/m) and a 25 mm birdcage resonator. Mice were anaesthetised with 1.5% isoflurane and kept at 37 °C. Electrocardiogram (ECG) and respiration were supervised with electrodes (Klear-Trace; CAS Medical Systems, Branford) and a pneumatic pillow, respectively. Vital functions were monitored by a M1025 system (SA Instruments, Stony Brook, NY, USA) and used to synchronise the cardiac magnetic resonance data acquisition with cardiac and respiratory motion if necessary. Contrast agent (CA) during the cMRI scan was infused through a Vasofix Safety IV catheter (B. Braun Melsungen AG, Melsungen, Germany) inserted into the peritoneal cavity. For multiparametric cMRI analysis in one experimental session, first ECG- and respiratory-triggered gradient-echo cine movies were recorded in short and long axis orientation. Subsequently, a bolus of gadolinium-diethylenetriaminepentaacetic acid (Gd-DTPA) [0.2 mmol Gd-DTPA per kg body weight (BW)] was infused through the peritoneal catheter for acquisition of post-contrast T1 maps and late gadolinium enhancement (LGE) images. For the latter, an ECG- and respiratory-gated segmented fast gradient-echo sequence with steady state precession (FISP) was used with the following parameters: echo time (TE) = 1.2 ms, 128 segments, repetition time (TR) = 6–8 ms depending on the heart rate, acquisition time (TAcq) ≈ 1 min, slice thickness (ST) = 1 mm, field of view (FOV) = 30 × 30 mm^2^, matrix size (MS) = 128 × 128, scan number (NS) = 2. Routinely, 8–10 short axis slices were required for complete coverage of the LV. The entire scanning protocol took around 80 min and was well tolerated by all animals, which recovered within 2 min from anaesthesia. The peritoneal catheter was directly removed after the cMRI scan while the mice were still under anaesthesia. Images were acquired at day 3 after induction of anaemia and in time-matched shams, respectively, as well as 24 h after AMI, using the Bruker microimaging unit Micro 2.5 with actively shielded gradient sets (1.5 T/m) and a 25 mm birdcage resonator.

For evaluation of functional parameters end-diastolic volume (EDV) end-systolic volume (ESV) and stroke volume (SV), ventricular demarcations in end-diastole and end-systole were manually drawn with the ParaVision region-of-interest (ROI) tool (Bruker, Rheinstetten, Germany). All regional data were derived from a midventricular slice. An in-house developed software module based on LabVIEW (National Instruments, Austin, USA) divided the LV into 200 equivalent sectors starting from the upper insertion point of the right ventricle, as described [6].

### RBC transfer model into isolated hearts with global ischaemia/reperfusion

A Langendorff model of global ischaemia/reperfusion [44] was modified as follows: anaemic and sham mice served as donors for blood samples, which were transferred at the beginning of 40 min global ischaemia into a recipient isolated WT heart. For a total duration of 40 min of global ischaemia, whole blood or RBC suspension was not washed out. Afterwards, hearts were subjected to 2 h of reperfusion. These experiments served as a bioassay to monitor RBC effects on LV function and coronary flow. In a separate set of mice RBC suspensions of anaemic or sham mice were incubated with the NO donor diethylamine NONOate sodium salt hydrate (DEA NONOate; 20 µmol/L, Sigma Aldrich, St Louis MO, USA) for 30 min [29] or with the NOS inhibitor NG-nitro-l-arginine methyl ester (L-NAME, 0.1 mmol/L, Sigma Aldrich, St Louis MO, USA) for 20 min at 37 °C before starting with the global ischaemia as previously described. To address the antioxidative pathway in this pathophysiological condition, we used the antioxidative compound NAC and pretreated anaemic and sham wild-type mice with 1% NAC in the drinking water for 4 weeks. Afterwards the RBCs from NAC-treated mice were isolated as described above and tested in the isolated heart [45].

### Statistics

Unless otherwise noted, results are expressed as the means ± standard deviation (SD). Multiple comparisons were made using two-way ANOVA followed by Sidak’s post hoc tests. One-way ANOVA (with Bonferroni´s correction for comparison of multiple means) or, where appropriate, equivalent non-parametric test (Dunn/Kruskal–Wallis multiple comparison) was used for comparison of serum/plasma parameters, blood pressure and cardiac functional parameters between three and more groups. For comparison of two groups, paired or unpaired Student’s *t* test was used. Shapiro–Wilk and Kolmogorov–Smirnov tests were employed for normality test before using analysis of variance or Student’s *t* test. Categorical variables were analysed using the chi-square test and were reported as frequencies and percentages. Statistical significance was tested using GraphPad Prism version 6.05. (Graphpad Software Inc., La Jolla, CA, US) or SPSS 23 (IBM, Armonk, NY, US). Only groups with similar variances were statistically compared. Outliers were identified by analysing box and whiskers plots according to Tukey. Normal distribution was tested by the D’Agostino–Pearson test. *p* values were considered as statistically significant and are provided either in the figures or tables. For in vivo experiments, the sample size was calculated using G*Power 3.1. A priori analysis with *t* test for two independent groups.

## Results

### Anaemia induces left ventricular functional compensation

**Fig. 1:**
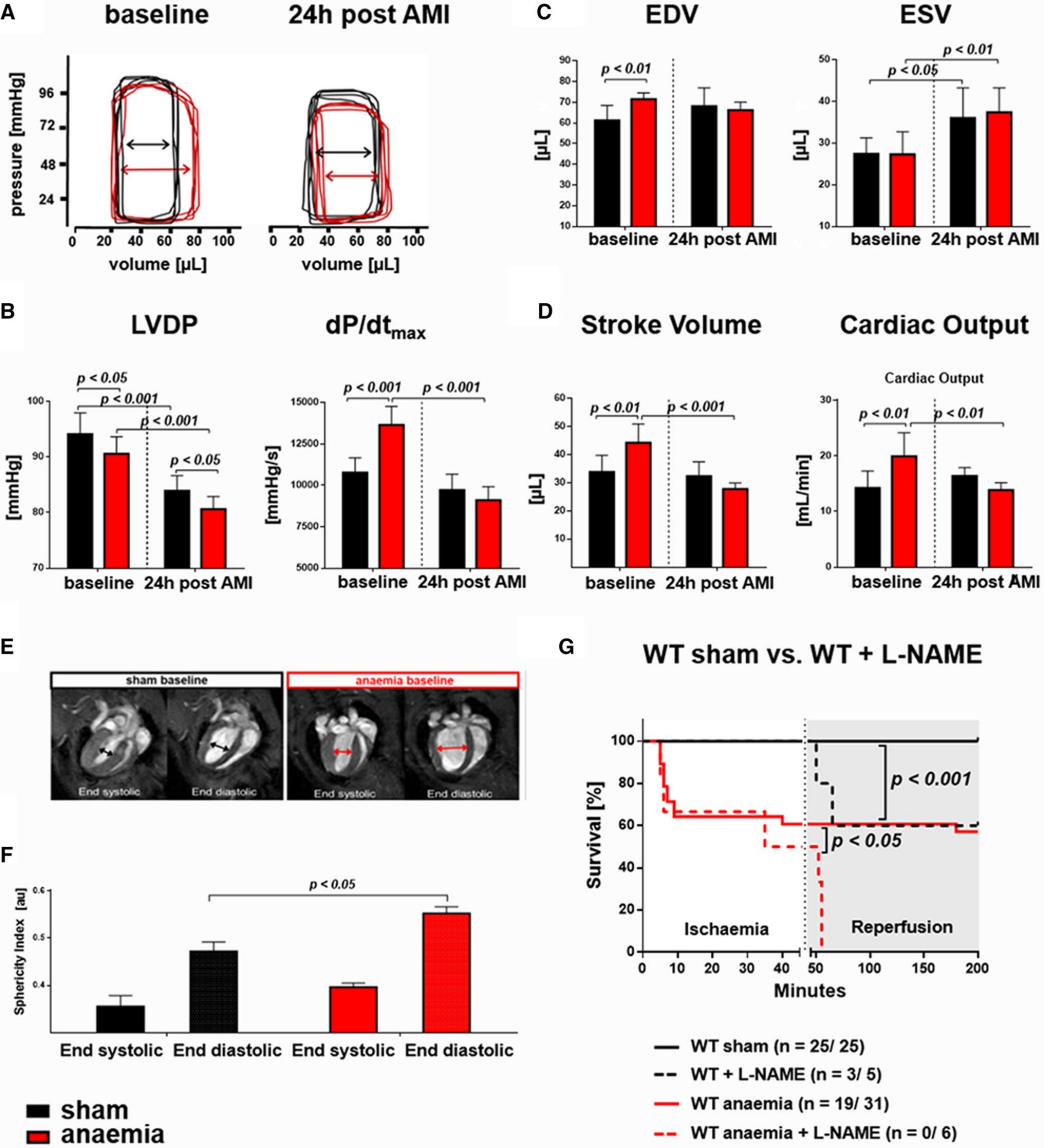
Anaemia-induced LV functional compensation. **a** Examples of non-calibrated original pressure–volume loops at baseline and 24 h post AMI with a rightward displacement in anaemic mice. **b** At baseline, left ventricular pressure (LVDP) was lower in anaemic mice while maximum rate of pressure rise (d*P*/d*t*_max_) was increased, and both were decreased post AMI. **c**, **d** In echocardiographic analysis, anaemic mice were characterised by an increase in end-diastolic volume (EDV), stroke volume (SV), and cardiac output (CO), while end-systolic volume (ESV) remained constant. In AMI, these compensatory differences were abrogated with an additional increase in ESV. **e** Representative images from cardiac magnetic resonance tomography imaging (cMRI) depicting a spherical reshaping of LV in anaemia **f** with increased baseline sphericity index of LV in anaemia. **g** Pharmacologic inhibition of eNOS in WT mice with *N*(ω)-nitro-l-arginine methyl ester (L-NAME) reduced survival post AMI. Data are mean ± SD from *n* = 6–10 (**b**), *n* = 8 (**c**), *n* = 6 (**f**) mice/group

**Fig. 2:**
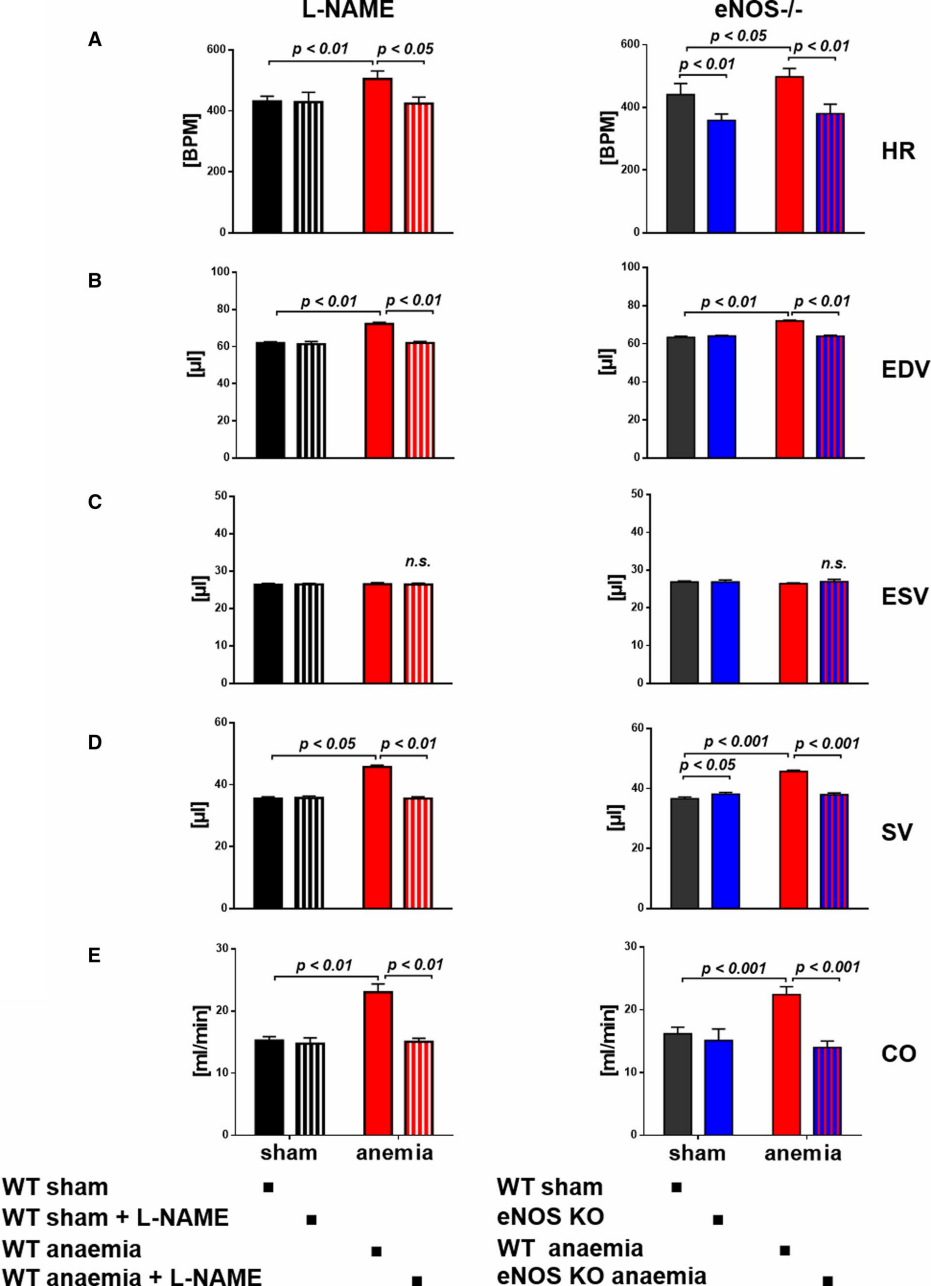
eNOS-inhibition abrogates LV functional compensation in anaemia. Anaemia induced increases in **a** heart rate (HR), **b** end-diastolic volume (EDV), **c** end-systolic volume (ESV), **d** stroke volume (SV) and **e** cardiac output (CO), which were abrogated after pharmacologic inhibition or genetic deletion of eNOS. Data are mean ± SD from *n* = 6 (L-NAME) and *n* = 5 (eNOS^−/−^) mice/group

Increased LV function compensated for anaemia at baseline in both the subacute and the chronic models of anaemia (Fig. 1, Online Resource Table S1 and S2). Anaemic mice had a rightward displacement of pressure–volume loops at baseline; left ventricular developed pressure (LVDP) was slightly reduced, while end-systolic volume (ESV) was maintained with higher maximum rate of LV pressure increase (Fig. 1a–c). These changes in LV function were accompanied by reshaping of the LV with increased sphericity (Fig. 1d–f) and a trend to increased eNOS expression (1.0 ± 0.4 versus 1.3 ± 0.6 eNOS normalised to housekeeping gene GAPDH, sham versus anaemia, *n* = 11/12, *p* = 0.25). The NOS inhibitor *N*(ω)-nitro-l-arginine methyl ester (L-NAME), administered prior to anaemia, prevented the anaemia-induced increases in heart rate (HR), end-diastolic volume (EDV), stroke volume (SV) and cardiac output (CO) and the same response was seen in eNOS^−/−^ mice (Fig. 2).

### Superimposition of reperfused AMI on anaemia is associated with increases in mortality: role of eNOS

More than one third of anaemic mice died from reperfused AMI as compared to none in the sham group. In anaemic and sham mice, which survived AMI, infarct sizes 24 h post AMI were not different, as evidenced by TTC staining (49 ± 7% in sham vs. 50 ± 10% IS/AAR in anaemic WT mice, *p* > 0.05, *n* = 8/8) or late gadolinium enhancement in cMRI analysis (24 ± 3% in sham vs. 24 ± 5% late gadolinium enhancement/LV in anaemic WT mice, *p* > 0.05, *n* = 6/6).

Anaemic mice had reduced CO, due to a lack of further increase in HR and a reduced SV (Fig. 1c, d, Online Resource Table 1B). Mortality increased with decreasing Hb-levels and was further increased with L-NAME in drinking water starting 4 days prior to ischaemia (Fig. 1G). These data were confirmed with application of either 2-ethyl-2-thiopseudourea (ETU) during ischaemia in WT mice (data not shown) or in eNOS^−/−^ mice: all sham/non-anaemic eNOS^−/−^ survived reperfused AMI, while five out of seven eNOS^−/−^ with anaemia died.

Most of the differences in LV function between anaemic and sham mice seen 24 h after AMI were already detectable 4 h post AMI. One week after reperfused AMI, Hb was almost restored but there was still a trend towards reduced LV function and persistent LV reshaping (data not shown). The eNOS-dependent cardiac compensation of anaemia was comparable in subacute and chronic anaemia.

### Circulatory compensation of anaemia is eNOS dependent

**Fig. 3:**
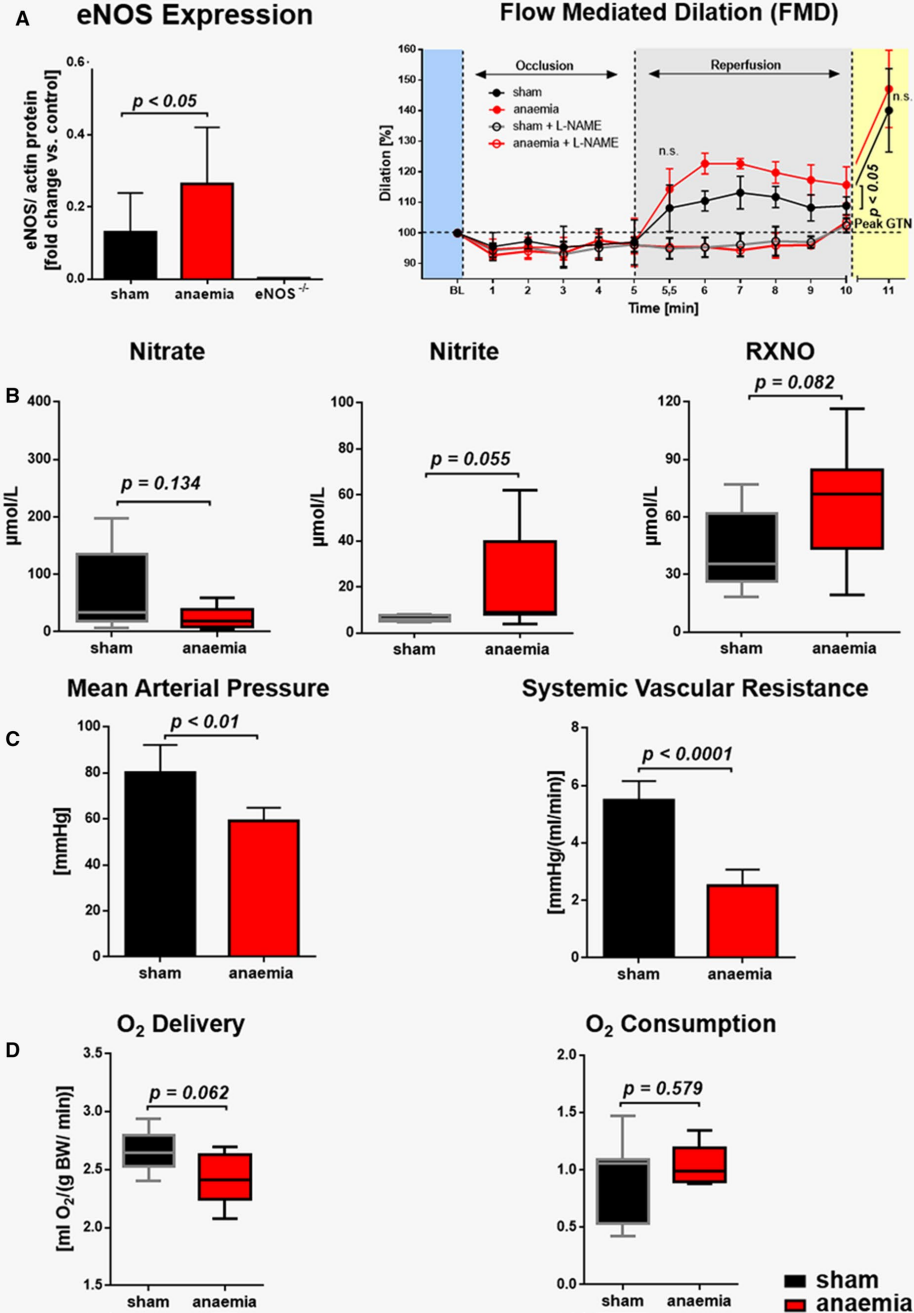
Increased vascular eNOS activity mediates circulatory adaptation to anaemia. **a** eNOS expression in the aorta was increased in anaemic mice with consecutively enhanced flow-mediated dilation (FMD) while the response to glycerol trinitrate (GTN) was unaffected. Compensatory increases in FMD were abrogated by eNOS inhibition with *N*(ω)-nitro-l-arginine methyl ester (L-NAME). **b** Oxidised and nitrosated NO metabolites in the aortic wall. **c** Mean arterial pressure and systemic vascular resistance were decreased in anaemia while **d** total body O_2_ consumption and O_2_ delivery were maintained. Data are mean ± SD from *n* = 3–8 (**a**, **b**) and *n* = 5–8 (**c**, **d**) mice/group

eNOS expression was significantly increased in the aorta of anaemic mice (Fig. 3a). This was also confirmed by an enhanced flow-mediated dilation (FMD) in anaemic mice, which was abolished by the NOS inhibitor L-NAME (Fig. 3a). An overall increased activity was also indicated by a trend towards increased nitrite and nitrosated NO in the aortic wall (Fig. 3b, Online Resource Table S3). Mice with anaemia were characterised by multiple compensatory changes, including decreases in blood pressure and systemic vascular resistance (Fig. 3c) to maintain total body oxygen delivery and consumption (Fig. 3d), furthermore, increases in RBC deformability and HR contributing to enhanced CO (Fig. 2a). Treatment with L-NAME attenuated the anaemia-induced decrease in mean arterial blood pressure (MAP) (Online Resource Table S4), suggesting a significant contribution of vascular eNOS to the compensation of anaemia.

### Anaemia leads to marked alterations in the circulating NO pool

**Fig. 4:**
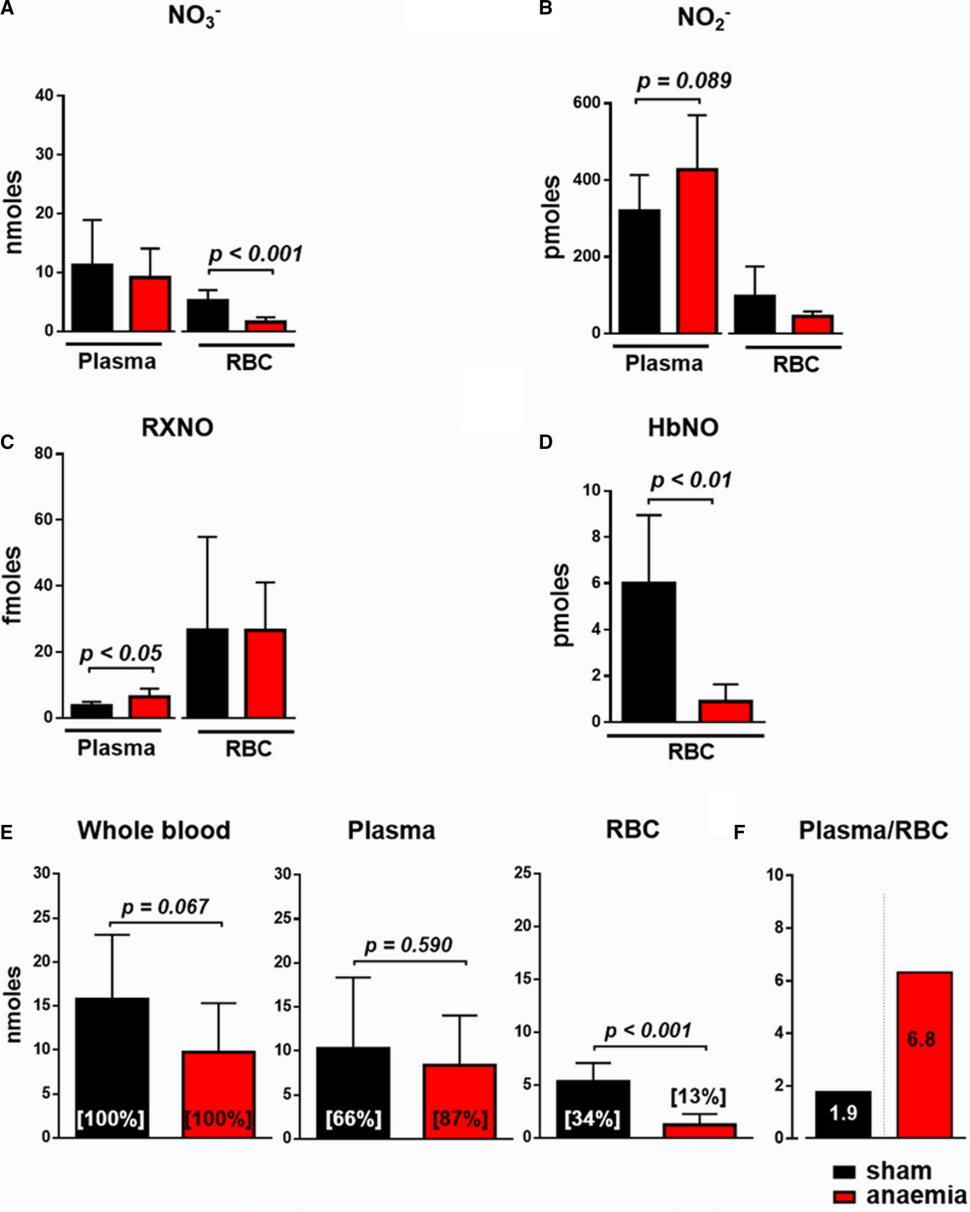
Anaemia decreases the circulating NO pool. The total amount of circulating nitrate (**a**), nitrite (**b**), nitrosated species (**c**) and NO-haem (**d**) in plasma and RBC from sham and anaemic mice. Proportional plot depicting the relative contribution of each metabolite to the total circulating NO pool. **e** Distribution of circulating NO pool in blood, calculated for 1 mL of blood and normalised to the haematocrit, and the percent of NO metabolites as a fraction of their total amount in 1 mL is given in brackets. **f** In moderate anaemia total, the circulating NO pool was reduced by 28%, along with a significant increase in the ratio of NO in plasma over that in RBC (1.9- to 6.8-fold). Data are mean ± SD from *n* = 6–8 (**a**–**d**) mice/group

In RBC from anaemic mice, the concentration of nitrate and the bioactive moiety of NO-haem were reduced by almost 30% (Online Resource Table S3). Given the decrease in haematocrit from 51 to 26% with a subsequent reduction of the total circulating RBC volume, the total amount of all oxidative and nitrosated NO metabolites in blood of anaemic mice was severely reduced (Fig. 4a–e). In the plasma of anaemic mice, the amounts of the oxidative metabolite nitrite and bioactive nitrosated species were increased (Fig. 4a–c). Since plasma volume was increased in the face of decreased RBC volume, there was on top of the overall reduced circulating NO pool a more than threefold increase of NO metabolites in plasma over that in RBC (Fig. 4f).

### RBC dysfunction in moderate anaemia

Repeated mild blood withdrawal of less than 15% of circulating blood volume per day and a stepwise average Hb decrease by less than 20 g/L on 3 consecutive days resulted in target levels of < 90 g/L Hb and changed the overall phenotype of mice. Normal lactate levels in plasma of anaemic mice largely excluded hypoxia secondary to moderate blood withdrawal. After consecutive blood withdrawal and volume replacement with saline, blood volume was unchanged, as evidenced by measurements with Evans blue dye. With induction of anaemia, several indexes of RBC morphology increased significantly or in part by trend such as, anisocytosis, rouleaux formation, red blood cell distribution width (RDW), mean corpuscular volume (MCV), mean corpuscular haemoglobin (MCH), and mean corpuscular haemoglobin concentration (MCHC) (Online Resource Table S5). Within 1 week after the last blood withdrawal, initial values of RBC morphology and Hb concentration were reestablished.

**Fig. 5:**
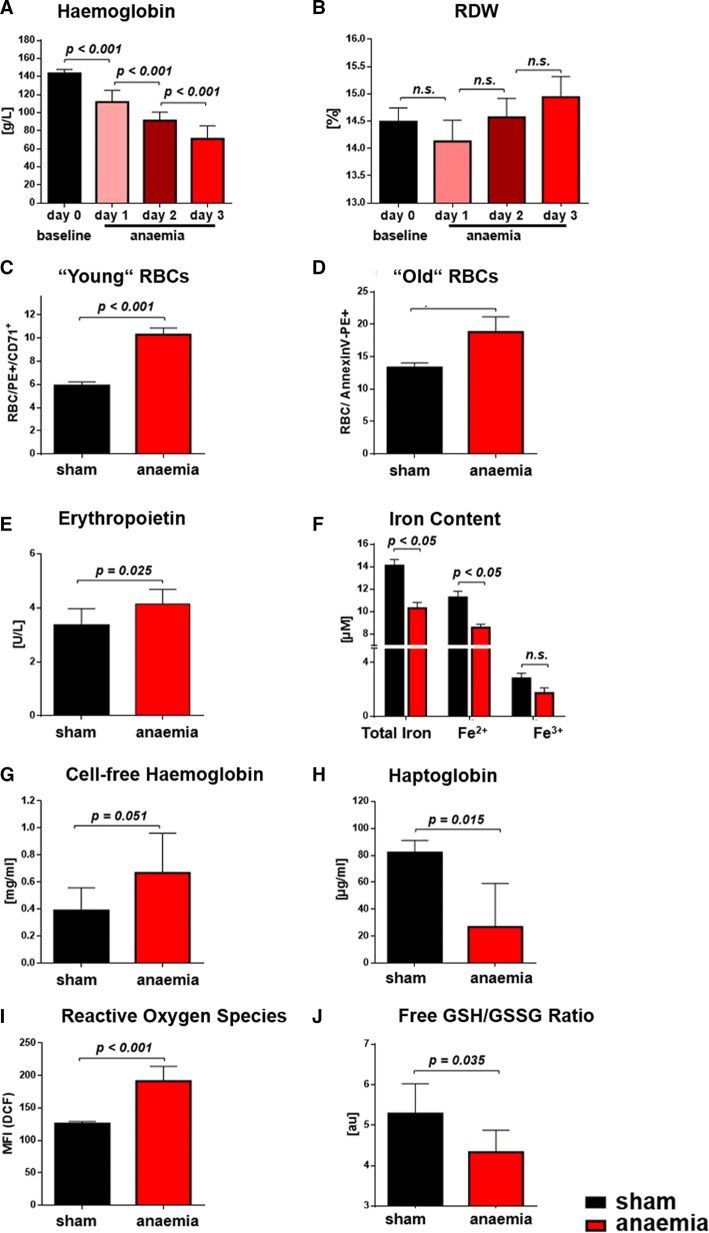
Moderate anaemia induces RBC dysfunction. **a**, **b** Changes in haemoglobin and red blood cell distribution width (RDW). In anaemia, the count of RBC labelled by either CD71+ (**c**) or Annexin V (**d**) was elevated. **e** Erythropoietin, **f** iron content, **g** cell-free Hb were increased, while **h** haptoglobin in plasma was decreased. **i**, **j** RBC of anaemic mice had elevated oxidative stress as reflected by increased levels of reactive oxygen species (ROS), while the free GSH/ GSSG ratio was decreased. Data are mean ± SD from *n* = 10 (**a**, **b**), *n* = 5 (**c**, **d**), *n* = 6–7 (**e**–**h**), *n* = 4–6 (**i**, **j**) mice/group

Anaemic mice had significantly decreased Hb (Fig. 5a) with a trend of an increased RDW (Fig. 5b). In line with this observation, several parameters reflected an accelerated turnover of RBC in anaemia: the number of young, CD71+ RBC (Fig. 5c) was increased, along with increased numbers of “old”/damaged, phosphatidylserine (PS^+^)-positive RBC (Fig. 5d), the latter being consistent with slightly increased levels of erythropoietin (Fig. 5e). Iron deficiency (Fig. 5f) and increased haemolysis became evident, as demonstrated by borderline increased plasma cell-free Hb concentrations (Fig. 5g) and reduced plasma haptoglobin (Fig. 5h). Reactive oxygen species (ROS) increased significantly in RBC of anaemic mice (Fig. 5i). Increased ROS formation was accompanied by an altered redox state with a decrease in the ratio of free to oxidised glutathione (GSH/GSSG, Fig. 5j), mainly due to an increase in oxidised glutathione (GSSG). A compensatory increase of RBC deformability was observed at low shear rates, as measured by ektacytometry (0.0001 ± 0.01 versus 0.003 ± 0.02 elongation index, sham versus anaemia, *n* = 5/5, *p* = 0.025).

### Mechanisms of RBC dysfunction and its effects on LV recovery after ischaemia/reperfusion

**Fig. 6:**
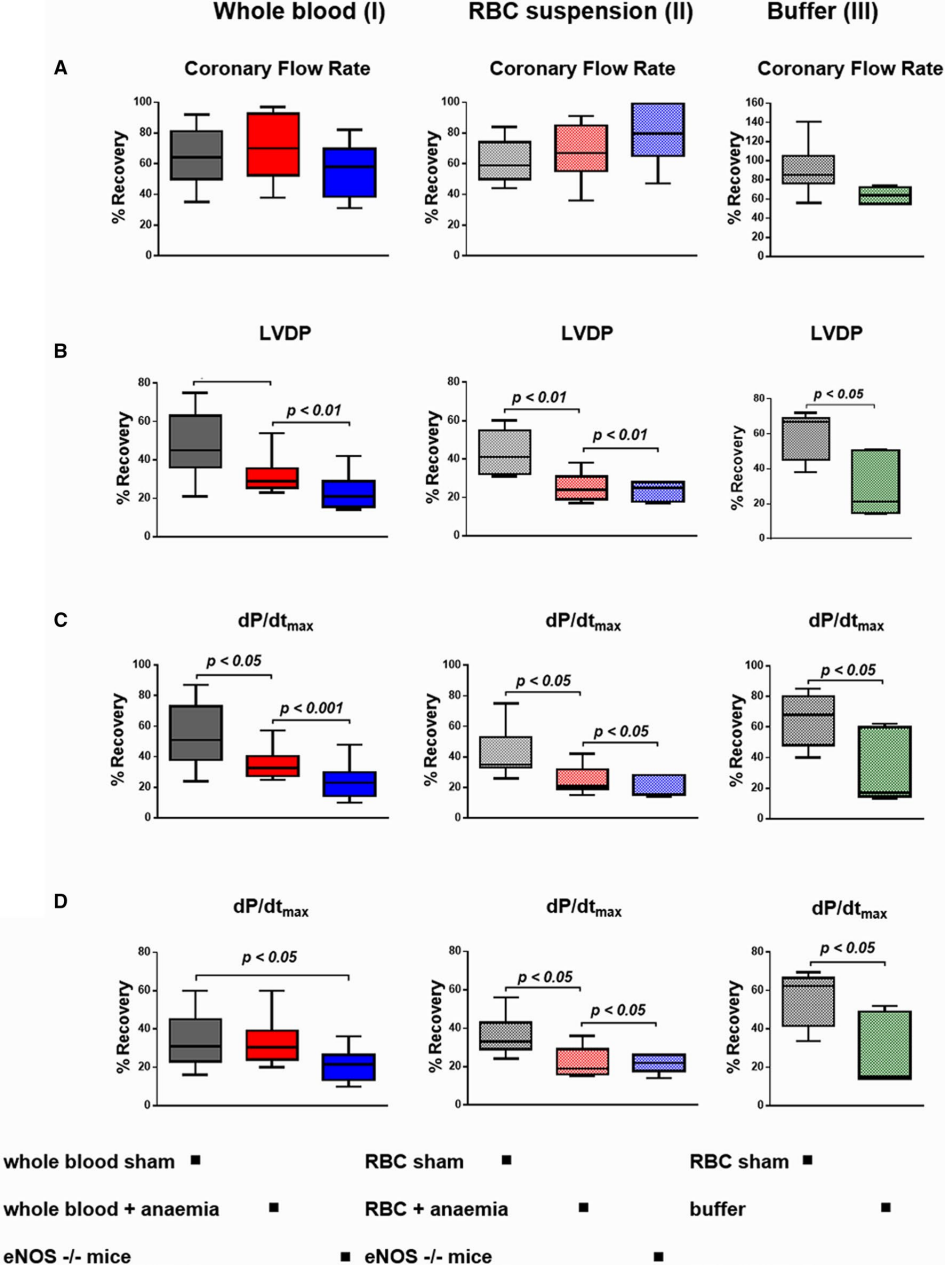
RBC from anaemic and eNOS deficient mice impair the recovery of LV function after ischemia/reperfusion. **a** Blood samples from anaemic WT mice and non-anaemic eNOS^−/−^ mice deteriorated in **b** left ventricular developed pressure (LVDP), **c** maximum rate of pressure increase (d*P*/d*t*_max_) and **d** (d*P*/d*t*_min_), as compared to sham mice (I). These effects were all specific for RBC (II). Control experiments revealed the cardioprotective effects of RBC from sham mice in recipient hearts after ischaemia/reperfusion (III). Data are mean ± SD from *n* = 7–11 (sham), *n* = 7–10 (anaemia), *n* = 6–8 (eNOS ^−/−^) and *n* = 5 (buffer) mice/group

To study whether RBC dysfunction in anaemia affects cardiac function, whole blood or re-suspended RBC from either anaemic or sham mice, respectively, were infused into isolated recipient murine hearts prior to global ischaemia/reperfusion; haematocrit was matched to exclude differences in oxygen delivery. As compared to incubation with saline buffer, samples of whole blood from sham WT mice significantly improved the recovery of LV function after ischaemia/reperfusion at a comparable coronary flow rate (Fig. 6, Online Resource Table S6A). The samples of whole blood from eNOS^−/−^ mice were characterised by deteriorated LV performance post ischaemia/reperfusion (Fig. 6). In a cell-specific approach, isolated and re-suspended RBC, from sham WT mice improved the recovery of LV function during reperfusion, while cardioprotection was severely diminished with RBC from anaemic mice (Fig. 6, Online Resource Table S6C). The deleterious effects of RBC from eNOS^−/−^ mice (Fig. 6b–d) together with the observation of a reduced NO bioactivity in RBC (Fig. 4d) and an increased ROS generation (Fig. 5i, j) suggest that eNOS uncoupling might contribute to RBC dysfunction in anaemia. This notion is mechanistically further supported by our findings that the NOS inhibitor L-NAME attenuated the deleterious effects of RBC from anaemic mice on LV function, while concomitant supplementation with the NO donor NONOate rescued the negative effects of these RBC (Online Resource Table S7). The redox imbalance in RBC from anaemic mice, in part driven by the uncoupling of RBC-eNOS, was further evidenced by slightly increased LVDP after treatment of anaemic RBC with the ROS inhibitor NAC (sham + NAC 35 ± 9 mmHg vs. anaemia + NAC 32 ± 6 mmHg).

### RBC dysfunction in ACS patients with anaemia

**Fig. 7:**
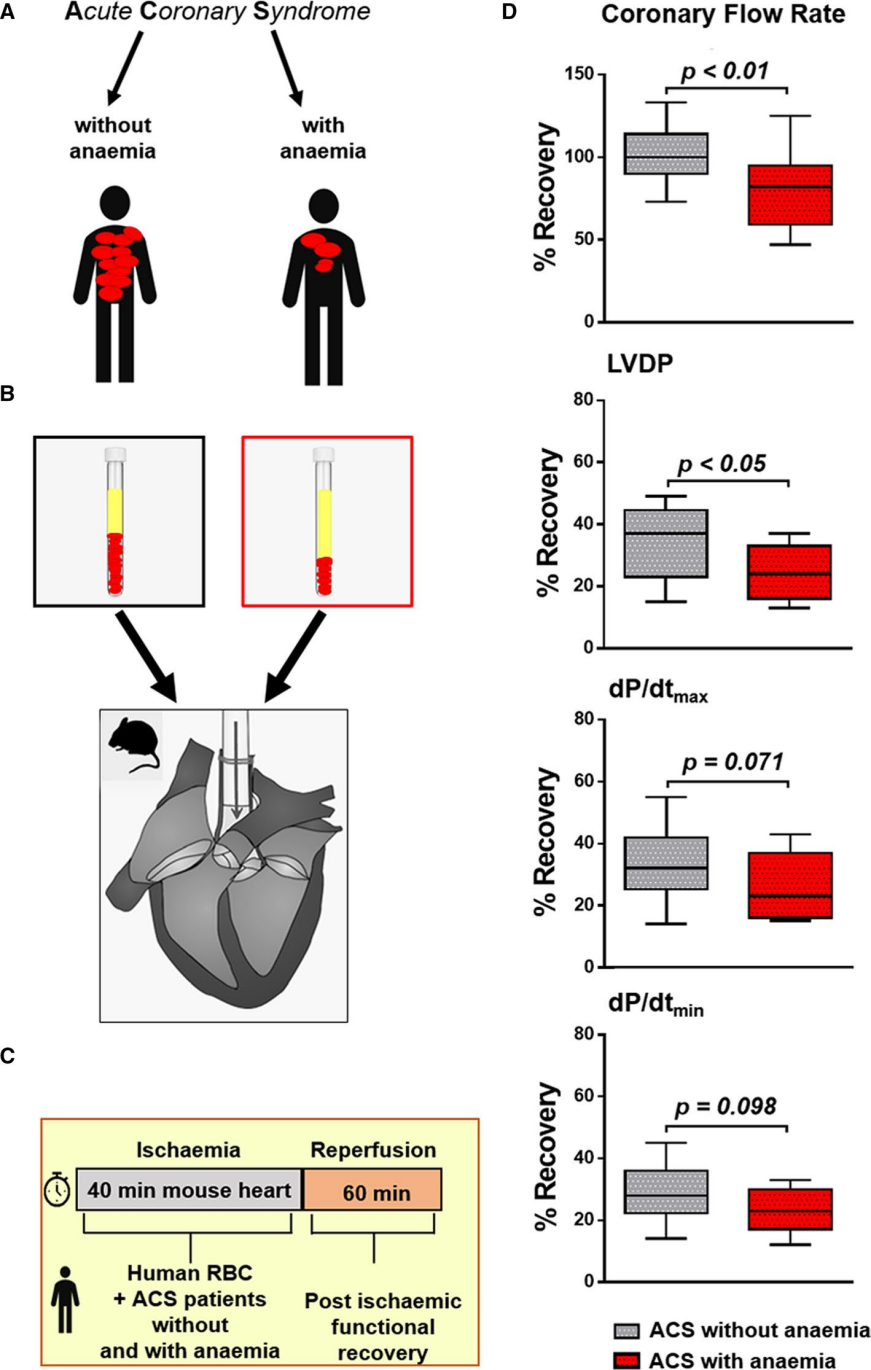
RBC from ACS patients with anaemia impair the recovery of LV contractile function after ischemia/reperfusion. Blood samples were taken from patients with acute coronary syndrome (ACS) at the time of admission, and the study cohort was analysed in relation to the presence or absence of anaemia (**a**). RBC were given into isolated mouse hearts prior to ischaemia/reperfusion (**b**). Cardiac contractile function was analysed at baseline and after ischaemia/reperfusion (**c**). LV function was reduced in hearts subjected to RBC from ACS patients with anaemia. Data are given in percent recovery of LV function from baseline (**d**). Data are mean ± SD from *n* = 14 (patients without anaemia), *n* = 15 (patients with anaemia)

To translate the concept of RBC dysfunction seen in mice with anaemia, we infused RBC taken on the day of admission from ACS patients with and without anaemia into the ex vivo mouse heart model of ischaemia/reperfusion with matched haematocrit (Hct). Presenting characteristics of these ACS patients exhibited typical profiles of cardiovascular risk factors, procedural characteristics, and contemporary medication (Online Resource Table S8). In ACS patients with anaemia, the analysis of blood parameters revealed moderated changes in Hb, Hct, iron with still normal saturation of transferrin, indices of reticulocytes, and RDW indicating RBC dysfunction as seen in mice models of moderate anaemia (Online Resource Table S9). RBC from anaemic patients lost their cardioprotective properties in the ex vivo model of ischaemia/reperfusion (Fig. 7, Online Resource Table S9). The magnitude of impairment in recovery of contractile LV function was comparable to that seen with RBC from WT with anaemia and eNOS^−/−^ mice (Fig. 6).

## Discussion

**Fig. 8:**
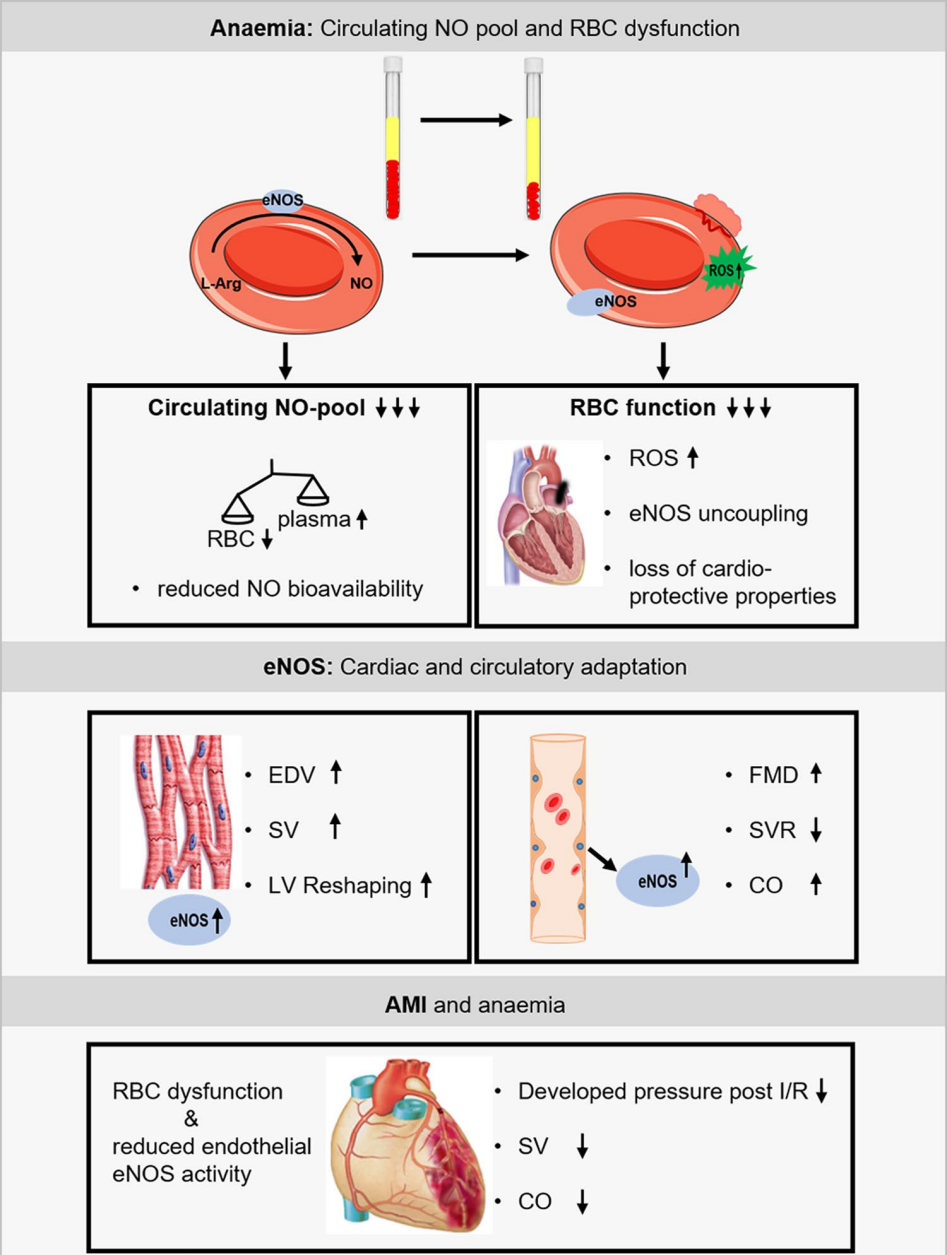
Graphical abstract for eNOS related-changes in anaemia. Moderate blood loss anaemia is associated with a reduced circulating nitric oxide (NO) pool and severe red blood cell (RBC) dysfunction. Vascular and cardiac endothelial nitric oxide synthase (eNOS) is crucial for the cardio-circulatory adaptation to anaemia. RBC dysfunction and a reduced endothelial eNOS activity contribute to left ventricular (LV) dysfunction and fatal outcome in acute myocardial infarction (AMI) superimposed on anaemia

Our data highlight major interrelated eNOS-dependent mechanisms in anaemia which might contribute to the adverse prognosis in AMI: (1) RBC dysfunction with reduced NO bioavailability, uncoupling of eNOS, increased ROS formation, reduced membrane integrity and increased NO scavenging by free plasma haemoglobin, all resulting in a reduction of the circulating NO pool, (2) compensatory enhancement of vascular and myocardial eNOS activity to mediate the cardio-circulatory adaptation to anaemia, (3) anaemia-associated RBC dysfunction together with endothelial eNOS dysfunction which may contribute to adverse outcomes in ischemic heart disease and AMI (Fig. 8).

### Models of moderate anaemia and RBC dysfunction

The degree of blood loss in mice with subacute and chronic anaemia was moderate and resembled that seen in ACS patients with anaemia. We performed repeated blood loss of only < 15% of the circulating blood volume per day with concomitant saline replacement. Signs of haemorrhage or significant volume shifts were excluded as arterial lactate concentration and the distribution of Evans blue dye remained within the normal range. Our findings may be extrapolated to other subtypes of anaemia such as in infectious diseases or nutritional disorders only with caution.

In our model of anaemia, enhanced turnover and dysfunction of RBC were apparent from a number of cellular and plasma markers of RBC integrity and function as shown in Fig. 5. Repeated moderate blood loss induced mild iron deficiency, as also frequently seen in anaemic patients [25, 34, 35]. All changes in blood count were reversible within 1 week. There were also signs of mild haemolysis as evidenced by increased cell-free haemoglobin and reduced haptoglobin, which might have affected the composition of the circulating NO pool. Others and we have shown that increased plasma levels of free Hb affect blood rheology and vascular vasodilator function through diminished NO availability [27], a mechanism that becomes even more prominent in sickle cell anaemia [18]. RBC are well equipped with a number of anti-oxidant defences, which primarily exist to keep Hb in its reduced (Hb-Fe^2+^) O_2_-binding form, among them most importantly the antioxidant GSH system. In RBC from anaemic mice, GSSG was increased and the ratio of free GSH to GSSG decreased, evidencing altered RBC redox state and together with the reduced levels of NO haem, suggesting decreased NO bioactivity.

### eNOS-related mechanisms of RBC dysfunction

RBC dysfunction affects the interaction of RBC with vascular and cardiac tissue; this notion is supported by the experiments with infusion of whole blood or washed RBC from anaemic mice into a recipient mouse heart with global ischaemia/reperfusion, where post-ischaemic recovery of LV function was impaired. Differences in oxygen supply were excluded by reconstituting RBC in both groups at identical haematocrit. The magnitude of effect was comparable to that seen with RBC from eNOS^−/−^ mice, which are devoid of cardioprotective RBC-eNOS. These findings are in line with the recent recognition that RBC can protect the heart from IR injury via the export of NO bioactivity [13]. An uncoupling of eNOS has been identified as major source of increased ROS production in RBC from diabetic patients, which not only attenuated the cardioprotection in isolated hearts with ischaemia/reperfusion, but also aggravated endothelial dysfunction [45, 47]. Indeed, in our studies, concomitant NOS inhibition attenuated and NO supplementation vice versa rescued the negative effects of RBC on LV function, supporting the notion of eNOS uncoupling with altered redox balance in RBC as one possible mechanism for RBC dysfunction in anaemia. We could also translate this concept to ACS patients with anaemia. Their RBC also impaired LV contractile function after ischaemia/reperfusion, and the loss in cardioprotection was comparable to that seen with RBC from anaemic mice. The exact mechanism of the export of cardioprotective NO bioactivity from RBC has not been identified definitely. Mice lacking ßCys93 S-nitrosylation exhibited greater cardiac injury and mortality in models of AMI pointing towards a significant role RBC-derived SNO-based vasoactivity in cardioprotection [46].

Due to the volume expansion of plasma in anaemia, the reduction of the total amount of RBC, and the decreased NO bioactivity within the remaining RBC, the overall circulating NO pool was reduced by one third, whereas the NO concentration in plasma was markedly increased over that in RBC. Increasing circulating NO pool through modulation of RBC function in patients with ischemic heart disease has been implicated as a novel target to compensate for microvascular dysfunction [14]. Given the high incidence of mild and moderate anaemia in patients with ACS [25, 34] these findings may have implications for patient management on intensive and coronary care units, since hospital-acquired anaemia due to repetitive blood withdrawal develops in nearly half of AMI hospitalizations, commonly in the absence of documented bleeding, and is associated with increased mortality [35]. Although there were no obvious differences between anaemic and non-anaemic ACS patients with respect to nutritional status we cannot differentiate, whether or not these findings are applicable to distinct types of anaemia.

### Role of vascular and cardiac eNOS to compensate for anaemia and AMI

We demonstrate circulatory compensation of anaemia by enhanced eNOS expression in the arterial wall and increased NO bioactivity as reflected by enhanced FMD, which was abrogated with pharmacological or genetic disruption of eNOS activity, and by the attenuation of the anaemia-induced drop of MAP by L-NAME. The anaemia-induced increase in CO led to a > 50% increase in blood flow velocity and thus calculated wall shear stress, which might have contributed to the enhanced eNOS activation. NO also contributed to the cardiac adaptation to anaemia with increased EDV and SV, and acute pharmacologic or chronic genetic inhibition of cardiac eNOS expression abrogated the anaemia-induced LV compensation. We and others have previously shown that NO derived from eNOS or nitrite reduction contributes to the regulation of coronary blood flow, LV function, [30, 32] the adaptation to myocardial ischaemia [21, 31, 37], and to a down- and rightward-displacement of the LV end-diastolic pressure–volume relation [22, 30], and early LV remodelling post AMI [20]. While the present experiments with pharmacologic NOS inhibitors might not exclude a role for inducible nitric oxide synthase (iNOS) or neuronal nitric oxide synthase (nNOS), the data obtained with eNOS^−/−^ mice clearly indicate a significant role of eNOS in the cardiac adaptation to anaemia. With superimposition of AMI, the eNOS-dependent cardiac and circulatory compensation were exhausted, and mortality was increased, as it was in wild-type mice with acutely induced eNOS dysfunction or in eNOS^−/−^ mice. While infarct sizes were not different, surviving anaemic mice had a loss of LV functional compensation. The more Hb was decreased, the more was mortality in anaemic mice increased after AMI. Similarly, the risk of death also increases in AMI patients at Hb levels below 90.0 to 80.0 g/L [3].

## Conclusions and clinical implications

We identified impaired eNOS as key to RBC dysfunction in anaemic mice and ACS patients. RBC dysfunction contributes to the deterioration of LV function post I/R. The upregulation of vascular and cardiac eNOS is crucial to compensate the reduced circulating NO pool in anaemia. Endothelial dysfunction induced through genetic or pharmacologic reduction of eNOS-activity abrogated the anaemia-induced cardio-circulatory compensation. Therefore, it is tempting to speculate that RBC dysfunction together with endothelial dysfunction might contribute to the impairment of LV function and outcome of AMI in anaemia. In ST-segment elevation myocardial infarction (STEMI) patient’s endothelial dysfunction resulting from increased age, hyperlipidaemia, arterial hypertension, diabetes mellitus, and chronic kidney diseases (CKD) is frequently present. AMI patients with diabetes or renal dysfunction (CKD III–V) have the worst prognosis; superimposition of anaemia more than doubles 10-year-mortality in these patients [41]. Understanding the complex role of anaemia in AMI is critical to counteract its adverse impact and to improve therapeutic strategies. Targeting not only reduced Hb levels, but more comprehensively the eNOS-related mechanisms of RBC dysfunction, and improving the cardio-circulatory adaptation may provide novel therapeutic approaches in patients with anaemia and superimposed AMI.

## Electronic supplementary material

Below is the link to the electronic supplementary material.

Supplementary file1 (PDF 692 kb)

## Data Availability

Further data available on request.
