# Supplementary material for: Anaemia is associated with severe RBC dysfunction and a reduced circulating NO pool: vascular and cardiac eNOS are crucial for the adaptation to anaemia
Source: Basic Res Cardiol. 2020 Jun 12;115(4):43. doi: 10.1007/s00395-020-0799-x (PMC7293199; doi:10.1007/s00395-020-0799-x)
Supplement: Supplementary file 1 — Supplementary file1 (PDF 692 kb) [file 395_2020_799_MOESM1_ESM.pdf]

Anaemia is associated with severe RBC dysfunction and a reduced circulating NO pool: vascular and cardiac eNOS are crucial for the adaptation to anaemia

Running title: *Wischmann et al.;* anaemia and myocardial infarction

Basic Research in Cardiology

Patricia Wischmann*, MD[1,2,3], Viktoria Kuhn*, MD[1,2,3], Tatsiana Suvorava, PhD[1,2,3], Johanna M. Muessig, MD[1,2,3], Jens W. Fischer, PhD[1,4], Brant E. Isakson, PhD[5], Sebastian M. Haberkorn, MD[1,2,6], Ulrich Flögel, PhD[1,2,6], Jürgen Schrader, MD[1,6], Christian Jung, MD[1,2,3], Miriam M. Cortese-Krott, PhD[1,2,3], Gerd Heusch, MD[7], Malte Kelm, MD[1,2,3]

[1] CARID, Cardiovascular Research Institute Duesseldorf; [2] Division of Cardiology, Pulmonary Diseases and Vascular Medicine, University Hospital of Duesseldorf, Germany; [3] Cardiovascular Research Laboratory, Medical Faculty, Heinrich-Heine University, Duesseldorf, Germany; [4] Department of Pharmacology and Clinical Pharmacology, Heinrich-Heine University, Duesseldorf, Germany; [5] Department of Molecular Physiology and Biological Physics, Robert M. Berne Cardiovascular Research Centre, University of Virginia School of Medicine, Charlottesville, Virginia, USA; [6] Department of Molecular Cardiology, Heinrich Heine University, Duesseldorf, Germany; [7] Institute for Pathophysiology, West German Heart and Vascular Centre, University of Essen Medical School, Essen, Germany

Supplemental material

Table of content **Pages**

Supplemental tables **3**

- Tab. 1: Haemodynamics in subacute anaemia at baseline and post AMI 3
- Tab. 2: Haemodynamics in chronic anaemia at baseline and post AMI 4
- Tab. 3: Circulating Nitric oxide (NO) metabolites in subacute anaemia 5
- Tab. 4: Blood count in subacute (d3) anaemia 6
- Tab. 5: Red blood cell (RBC) effects on left ventricular (LV) function 7
- Tab. 6: Effects of NO on RBC function 10
- Tab. 7: Patient Characteristics of human RBC Donors 12
- Tab. 8: Patient laboratory parameters of human RBC Donors 14
- Tab. 9: Effects of RBC from patients with ACS on left ventricular (LV) function 15

Supplemental Tables

<u>Table S1</u>: Haemodynamics in subacute anaemia at baseline and post AMI

	Tab. 1A Baseline					Tab. 1B post AMI				
**Circulatory and cardiac function**	**sham**		**anaemia**		***p-value vs sham***	**sham**		**anaemia**		***p-value vs sham***
**Echocardiography analysis**		**± SD**		**± SD**			**± SD**		**± SD**	
Heart rate [BPM]	420	± 29	479	± 14	< 0.05	531	± 53 †	499	± 25	
Stroke volume [µL]	33.8	± 6.0	44.2	± 6.7	< 0.01	32.3	± 5.2	27.8	± 2.1 ††††	
Cardiac output [mL/min]	14.3	± 3.1	20.00	± 4.2	< 0.01	16.4	± 1.5	13.9	± 1.3 ††	
End-diastolic volume [µL]	61.3	± 7.1	71.5	± 3.0	< 0.01	68.1	± 8.9	66.2	± 3.8	
End-systolic volume [µL]	27.4	± 3.8	27.3	± 5.4		36.0	± 7.2 †	37.4	± 5.8 ††	
**Invasive catheterization**		**± SD**		**± SD**			**± SD**		**± SD**	
Systolic blood pressure [mmHg]	99.5	± 11.6	83.8	± 3.6	< 0.05	80.0	± 4.7 †	72.8	± 6.4	< 0.05
Diastolic blood pressure [mmHg]	65.7	± 12.7	55.6	± 3.1	< 0.05	47.0	± 5.4 †	44.3	± 8.2 †	
LV developed pressure [mmHg]	94.1	± 3.8	90.6	± 3.0	< 0.05	83.9	± 2.7 ††††	80.6	± 2.2 ††††	< 0.05
dP/dt_{max} [mmHg/s]	10778	± 891.5	13649	± 1127	< 0.001	9425	± 952.8	9114	± 814.8 ††††	
dP/dt_{min} [mmHg/s]	-8638	± 355.3	-8571	± 714.9		-7316.6	± 717.9 †	-6840.0	± 541 †	

Data are mean ± SD; echocardiography: n = 8-12 mice/group; pressure-volume-loop: n= 6 mice/group. $p < 0.05$: † vs. baseline, $p < 0.01$: †† vs. baseline, $p < 0.0001$: †††† vs. baseline. BPM = beats per minute, dP/dt_{max} = maximum rate of pressure increase, dP/dt_{min} = maximum rate of pressure decreases, LV = left ventricular.

Table S2: Haemodynamics in chronic anaemia at baseline and post AMI

	baseline									post 24h AMI					
	sham				chronic anaemia					sham			chronic anaemia		
Cardiac function parameter	Baseline (d0)	± SD	Pre-AMI (d42)	± SD	Baseline (d0)	± SD	Pre- AMI (d42)	± SD	*p-Value* *vs baseline, vs sham*	24 h post AMI (d44)	± SD	*p-Value* *vs baseline, vs pre-AMI*	24h post AMI (d44)	± SD	*p-Value* *vs baseline, vs pre-AMI*
**Heart rate [BPM]**	421	± 30	450	± 10	427	± 19	481	± 47	< 0.05	521	± 30	< 0.05, < 0.05	510	± 40	< 0.05
**Stroke Volume [µL]**	32.6	± 2.5	31.8	± 2.8	34.3	± 1.1	47.9	± 3.9	< 0.05, < 0.05	26.3	± 1.4	< 0.05, < 0.05	22.8	± 3.4	< 0.05, < 0.05
**Cardiac Output [mL/min]**	13.7	± 1.7	14.3	± 1.5	14.6	± 0.7	23.0	± 2.4	< 0.05	13.7	± 1.1		11.7	± 2.2	< 0.05, < 0.05
**End-diastolic volume [µL]**	59.4	± 3.7	58.1	± 4.5	61.0	± 2.3	73.5	± 4.1	< 0.05, < 0.05	62.2	± 3.6		62.7	± 5.8	< 0.05, < 0.05
**End-systolic volume [µL]**	26.8	± 1.4	26.2	± 1.8	26.7	± 1.6	25.6	± 1.9		35.9	± 2.3	< 0.05, < 0.05	39.9	± 4.9	< 0.05, < 0.05

Data are reported as mean ± SD of 9-10 mice/group. BPM = beats per minute.

<u>Table S3</u>: Circulating NO metabolites in subacute anaemia

Metabolites	Concentration	Wild type sham	Wild type anaemia	*p-value vs. Wild type sham*
Erythrocytes				
Nitrate	µM	10.10 ± 3.19	6.24 ± 3.18	
Nitrite	nM	193.6 ± 178.2	180.4 ± 58.65	
RXNO	nM	55.32 ± 58.38	85.51 ± 60.34	
NO haem	nM	11.88 ± 5.84	3.27 ±2.67	<0.01
Plasma				
Nitrate	µM	21.97 ± 15.96	12.41 ± 6.68	
Nitrite	nM	671.4 ± 274.4	547.0 ± 206.6	
RXNO	nM	7.71 ± 2.43	9.59 ± 4.06	
Aorta				
Nitrite	µM	6.24 ± 1.24	22.06 ± 21.39	p = 0.055
Nitrate	µM	69.22 ± 70.62	24.94 ± 19.44	
RXNO	µM	41.46 ± 20.73	66.74 ± 30.79	p = 0.082

Data are shown as means ± SD from 5-9 mice/group; RXNO = nitrosation products; NO-haem = nitrosyl haem.

Table S4: Blood count in subacute (d3) anaemia

Blood count	d0 baseline	± SD	d3 subacute anaemia	± SD	*p-value vs baseline*
Haemoglobin [g/L]	143.5	± 4.0	80.9	± 14.0	< 0.05
Haematocrit [proportion]	0.44	± 0.03	0.25	± 0.04	< 0.05
RBC count [10^{12}/L]	8.9	± 0.3	4.9	± 0.9	< 0.05
MCV [fL]	49.8	± 0.4	51.8	± 1.3	< 0.05
MCH [pg]	16.2	± 0.4	17.8	± 0.8	< 0.05
MCHC [g/dl]	32.3	± 0.7	34.1	± 0.5	< 0.05
RDW [%]	14.5	± 0.3	14.9	± 0.4	
WBC count [10^{9}/L]	6.9	± 1.3	4.7	± 1.3	
Lymphocytes [10^{9}/L]	5.2	± 1.2	2.9	± 1.1	
Monocytes [10^{9}/L]	0.2	± 0.1	0.2	± 0.1	
Granulocytes [10^{9}/L]	1.5	± 0.3	1.3	± 0.7	
PLT count [10^{9}/L]	1281.8	± 80.4	1686.8	± 136.2	
LDH [U/L]	103.5	± 47.0	243.8	± 81.7	< 0.05
Lactate (arterial) [mmol/L]	2.77	± 0.5	2.52	± 0.5	
Lactate (venous) [mmol/L]	2.71	± 0.7	2.58	± 0.5	
pH (arterial)	7.38	± 0.01	7.42	± 0.02	
pH (venous)	7.37	± 0.04	7.37	± 0.03	
pO_2 (arterial) [mmHg]	99.41	± 3.9	94.2	± 4.7	
pO_2 (venous) [mmHg]	54.37	± 7.0	47.46	± 3.7	
SaO_2 (arterial) [%]	96.7	± 3.1	97.14	± 2.3	
SaO_2 (venous) [%]	63.43	± 10.9	54.9	± 5.4	

Data are means ± SD from n = 5-13 mice/group; d = day; MCH = mean corpuscular haemoglobin; MCHC = mean corpuscular haemoglobin concentration; MCV = mean corpuscular volume; PLT = platelet; RBC = red blood cell; RDW = red blood cell distribution width; WBC = white blood cell; LDH = Lactate dehydrogenase; pO_2 = partial oxygen pressure; SO_2 = oxygen saturation.

<u>Table S5</u>: Effects of whole blood (A) saline buffer (B), and RBC (C) on left ventricular (LV) function in isolated recipient hearts with ischaemia/reperfusion

TableS5A

**Condition**	**Whole blood**	**sham**	**± SD**	**anaemia**	**± SD**	***p-value vs sham***	**eNOS$^{-/-}$**	**± SD**	***p-value vs sham***
	Coronary flow [mL/min/g]	2.94	± 0.60	2.35	± 0.47		3.05	± 0.50	
	LV developed pressure [mmHg]	84.36	± 14.71	80.60	± 10.80		80.75	± 6.27	
	dP/dt_{max} [mmHg/s]	3697	± 673.3	3212	± 532.0		3433	± 418.1	
**Baseline**	dP/dt_{min} [mmHg/s]	-2825	± 501.2	-2279	± 420.5	< 0.05	-2367	± 350.2	
	Coronary flow [mL/min/g]	1.91	± 0.71	1.69	± 0.64		1.69	± 0.59	
	LV developed pressure [mmHg]	38.64	± 12.36	24.90	± 5.26	< 0.01	18.75	± 7.44	< 0.001
**60 min**	dP/dt_{max} [mmHg/s]	1915	± 596.0	1099	± 249.9	< 0.001	808.9	± 409.0	< 0.0001
**Recovery**	dP/dt_{min} [mmHg/s]	-966.7	± 366.1	-695.5	± 136.0	< 0.05	-498.6	± 206.3	< 0.01
	Coronary flow [% Recovery]	64.82	± 18.55	71.50	± 21.00		56.00	± 18.03	
	LV developed pressure [% Recovery]	46.91	± 16.84	31.70	± 9.25	< 0.05	23.50	± 9.55	< 0.01
**%**	dP/dt_{max} [% Recovery]	53.82	± 19.77	35.00	± 9.60	< 0.05	24.00	± 12.24	< 0.001
**Recovery**	dP/dt_{min} [% Recovery]	34.64	± 13.67	32.70	± 11.37		21.13	± 8.44	< 0.05

Table S5B

Condition	Buffer	buffer control	± SD	RBC of sham	± SD	*p-value vs buffer*	RBC of anaemia	± SD	*p-value vs buffer*
**Baseline**	Coronary flow [mL/min/g]	3.07	± 0.66	2.00	± 0.45		2.71	± 0.90	
	LV developed pressure [mmHg]	90.20	± 13.07	77.71	± 14.81		84.13	± 15.65	
	dP/dt_{max} [mmHg/s]	3752	± 426.8	3254	± 563.3		3381	± 647.2	
	dP/dt_{min} [mmHg/s]	!	± 491.2	-2445	± 685.3		-2681	± 564.5	
**60 min Recovery**	Coronary flow [mL/min/g]	2.00	± 0.66	1.89	± 0.80		2.05	± 0.74	
	LV developed pressure [mmHg]	28.80	± 20.84	44.71	± 13.92		37.25	± 11.59	
	dP/dt_{max} [mmHg/s]	1318	± 1094	2072	± 699.9		1714	± 606.6	
	dP/dt_{min} [mmHg/s]	-862.6	± 673.9	-1321	± 457.1		1045	± 357.9	
**% Recovery**	Coronary flow [% Recovery]	63.80	± 8.8	91.74	± 26.98		78.63	± 24.04	
	LV developed pressure [% Recovery]	30.20	± 18.73	58.00	± 14.07	< 0.05	44.88	± 12.82	
	dP/dt_{max} [% Recovery]	33.20	± 24.55	63.86	± 17.06	< 0.05	51.38	± 17.04	
	dP/dt_{min} [% Recovery]	28.20	± 19.11	55.14	± 14.21	< 0.05	39.38	± 12.06	

Table S5C

Condition	RBC Suspension	sham	± SD	anaemia	± SD	*p-value vs sham*	eNOS-/-	± SD	*p-value vs sham*
**Baseline**	Coronary flow [mL/min/g]	2.94	± 0.73	2.31	± 0.67		3.38	± 0.52	
	LV developed pressure [mmHg]	75.43	± 10.83	79.00	± 12.41		73.17	± 4.83	
	dP/dt_{max} [mmHg/s]	3007	± 504.9	3616	± 548.9	< 0.05	3333	± 282.3	
	dP/dt_{min} [mmHg/s]	-2286	± 364.5	-2494	± 467.9		-2150	± 204.2	
**60 min Recovery**	Coronary flow [mL/min/g]	1.78	± 0.60	1.59	± 0.62		2.76	± 1.05	
	LV developed pressure [mmHg]	31.43	± 7.68	19.71	± 6.40	< 0.01	23.66	± 4.98	
	dP/dt_{max} [mmHg/s]	1291	± 462.1	892.1	± 379.3		773.3	± 183.9	< 0.05
	dP/dt_{min} [mmHg/s]	-806.7	± 241.0	-556.3	± 204.7	< 0.05	-453.5	± 70.07	< 0.01
**% Recovery**	Coronary flow [% Recovery]	61.00	± 13.77	68.14	± 19.22		79.17	± 20.16	
	LV developed pressure [% Recovery]	42.43	± 11.76	25.14	± 7.47	< 0.01	23.50	± 4.85	< 0.01
	dP/dt_{max} [% Recovery]	44.00	± 16.82	24.43	± 9.33	< 0.05	23.67	± 6.74	< 0.05
	dP/dt_{min} [% Recovery]	35.71	± 10.70	22.71	± 7.68	< 0.05	21.50	± 4.55	< 0.05

In each table data are given for coronary flow and LV function at baseline, 60 min after recovery and recovery (%) in isolated hearts from healthy wild type mice loaded with **(A)** whole blood, **(B)** either saline buffer or a separate group of RBC pellet from sham and anaemic mice or **(C)** RBC suspension at the beginning of global myocardial ischaemia. Samples of whole blood or RBC were drawn from sham, anaemic WT mice or $eNOS^{-/-}$ mice. Data are means ± SD from n= 5-11 mice/group; (**A**) (n= 11 sham; n=10 anaemia; n= 8 $eNOS^{-/-}$, whole blood); **(B)** (n= 5 buffer; n= 7 sham; n=8 anaemia; RBC); **(C)** (n= 7 sham; n=7 anaemia; n= 6 $eNOS^{-/-}$; RBC); dP/dt_{max} = maximum rate of pressure increase; dP/dt_{min} = maximum rate of pressure decrease; LV = left ventricular.

Table S6: **Effects of RBC pre-treated with NOS Inhibitor L-NAME or NO donor NONOate on left ventricular (LV) function in isolated recipient hearts with ischaemia/reperfusion**

Table S6A

**Condition**	**RBC Suspension**	**sham+ L-NAME**	**± SD**	***p-value vs sham***	**anaemia+ L-NAME**	**± SD**	***p-value vs acute anaemia***
**Baseline**	Coronary flow [mL/min/g]	2.40	± 0.51		2.11	± 0.26	
	LV developed pressure [mmHg]	80.00	± 15.38		82.17	± 13.45	
	dP/dt_{max} [mmHg/s]	3709	± 684.9		3523	± 491.1	
	dP/dt_{min} [mmHg/s]	-2651	± 598.9		-2567	± 531.5	
**60 min Recovery**	Coronary flow [% Recovery]	1.95	± 0.26		2.42	± 0.50	< 0.05
	LV developed pressure [% Recovery]	46.67	± 17.88		37.17	± 16.13	< 0.05
	dP/dt_{max} [% Recovery]	2223	± 1036		1674	± 780.7	< 0.05
	dP/dt_{min} [% Recovery]	-1307	± 552.6		-1026	± 441.3	< 0.05
**% Recovery**	Coronary flow [% Recovery]	83.33	± 16.65		99.50	± 3.41	< 0.01
	LV developed pressure [% Recovery]	55.67	± 22.11		44.50	± 18.73	< 0.05
	dP/dt_{max} [% Recovery]	60.00	± 24.58		47.00	± 21.00	< 0.05
	dP/dt_{min} [% Recovery]	50.83	± 23.76		40.00	± 16.28	< 0.05

Tab. 6B

Condition	RBC Suspension	sham +NONOate	± SD	*p-value vs sham*	anaemia +NONOate	± SD	*p-value vs acute anaemia*
**Baseline**	Coronary flow [mL/min/g]	1.88	± 0.58	< 0.05	2.36	± 0.71	
	LV developed pressure [mmHg]	78.17	± 10.98		86.86	± 9.06	
	dP/dt_{max} [mmHg/s]	3098	± 384.4		3585	± 459.2	
	dP/dt_{min} [mmHg/s]	-2296	± 347.7		-2660	± 343.9	
**60 min Recovery**	Coronary flow [% Recovery]	1.62	± 0.51		2.11	± 0.74	
	LV developed pressure [% Recovery]	29.33	± 3.33		31.00	± 8.04	< 0.05
	dP/dt_{max} [% Recovery]	1204	± 195.7		1296	± 382.6	
	dP/dt_{min} [% Recovery]	-755.2	± 107.4		-896.3	± 382.1	
**% Recovery**	Coronary flow [% Recovery]	86.33	±9.40	< 0.01	89.14	± 8.14	< 0.05
	LV developed pressure [% Recovery]	38.50	± 8.87		36.41	± 10.37	< 0.05
	dP/dt_{max} [% Recovery]	40.17	± 10.80		36.29	± 9.53	< 0.05
	dP/dt_{min} [% Recovery]	34.00	± 9.72		31.71	± 10.29	

Data are given for coronary flow and LV function at baseline, 60 minutes after recovery and post-ischaemic recovery (%) in isolated hearts from healthy wild type mice loaded with sham or anaemic RBC suspension which was preincubated with **(A) L-NAME** or **(B) NONOate** at the beginning of global myocardial ischaemia. Data are means ± SD from n= 5-7 mice/group; (**A**) (n= 5 sham, n= 6 anaemia); (**B**) (n= 6 sham; n= 7 anaemia); p-value: Table S5C vs. sham and vs. anaemia. dP/dt_{max} = maximum rate of pressure increase; dP/dt_{min}= maximum rate of pressure decreases; LV= left ventricular.

Table S7: Presenting characteristics of patients with ACS

Characteristics	Patients (all) (n=29)		ACS without anaemia (n=14)		ACS with anaemia (n=15)		*p-value*
**Demographics**							
Age (years)	69	± 12	65	± 11	73	± 12	
Sex (male, %)	16	(55%)	8	(53.7%)	8	(53%)	
**Medical history**							
Hypertension (n, %)	23	(79%)	11	(79%)	12	(80%)	
Hyperlipidemia (n, %)	15	(52%)	9	(64%)	6	(40%)	
Diabetes mellitus (n, %)	8	(29%)	3	(21%)	5	(33%)	
Current smoker (n, %)	14	(48%)	5	(36%)	9	(60%)	
PAD (n, %)	7	(24%)	3	(21%)	4	(27%)	
Stroke (n, %)	1	(3%)	1	(7%)	0	(0%)	
Chronic kidney disease (n, %)							
CKD I-II (n, %)	16	(55%)	11	(79%)	5	(33%)	
CKD >III (n, %)	13	(45%)	3	(21%)	10	(67%)	
**Presenting characteristics**							
BMI	27.7	± 6.4	30.3	± 8.1	25.2	± 2.9	< 0.05
HR [bpm]	77	± 19	76	± 11	78	± 25	
Systolic BP	137	± 24	132	± 23	141	± 26	
Diastolic BP	74	± 11	74	± 13	74	± 10	
**Procedural characteristics**							
Primary PCI (n, %)	12	(41%)	5	(36%)	7	(47%)	
Microangiopathy (n, %)	4	(14%)	3	(21%)	1	(7%)	
1 vessel CAD (n, %)	3	(10%)	0	(0%)	3	(20%)	
2 vessel CAD (n, %)	1	(3%)	1	(7%)	0	(0%)	
3 vessel CAD (n, %)	21	(72%)	10	(71%)	11	(73%)	
EF (%)	29	49 ± 15	14	54 ± 14	15	44 ± 15	< 0.05
STEMI (n, %)	10	(34%)	6	(43%)	4	(27%)	
NSTEMI (n, %)	19	(66%)	8	(57%)	11	(73%)	
**Medications**							
Aspirin	24	(83%)	12	(86%)	12	(80%)	
ACEI/ARB	27	(93%)	14	(100%)	13	(87%)	
ß-Blocker	25	(86%)	12	(86%)	13	(87%)	
Lipid Lowering	23	(79%)	11	(79%)	12	(80%)	
P2Y12-Inhibtor	22	(76%)	11	(79%)	11	(73%)	
N(OAK)	8	(28%)	2	(14%)	6	(40%)	

Values are mean ± SD or are given as absolute values in %. ACS= acute coronary syndrome; PAD= peripheral arterial disease; CKD= chronic kidney disease; BMI= Body mass index; HR= heart rate; BP= blood pressure; PCI= percutaneous coronary intervention; CAD= coronary artery disease; EF= ejection fraction; STEMI= ST-elevation myocardial infarction; NSTEMI=

non ST-elevation myocardial infarction; ACEI= Angiotensin-converting-enzyme-inhibitor; ARB=Angiotensin-receptor-blocker; N(OAK)= (non) vitamin K antagonist oral anticoagulants.

Table S8: Laboratory parameters of patients with ACS on admission

**Laboratory chemical Parameters** [References]	**Patients (all) (n=29)**		**ACS without anaemia (n=14)**		**ACS with anaemia (n=15)**		***p-value***
Creatinine [mg/dl] [<=1.20]	1.14	± 0.57	0.87	± 0.23	1.39	± 0.68	< 0.05
GFR [mL/min] [90-140]	69.79	± 29.5	84.36	± 20.19	56.20	± 30.86	< 0.01
Haemoglobin [g/dl] [13.5-16.9]	11.83	± 2.68	14.16	± 1.31	9.66	± 1.52	< 0.01
Haematocrit [%] [40.0-49.4]	36.07	± 7.32	41.99	± 4.99	30.55	± 4.05	< 0.01
MCV [fL] [81.8-95.5]	93.29	± 5.6	93.26	± 4.32	93.31	± 6.73	
MCH [pg] [27.0-32.3]	30.03	± 2.28	30.64	± 1.28	29.47	± 2.86	
MCHC [g Hb/dl] [32.4-35.0]	32.16	± 1.58	32.80	± 0.84	31.55	± 1.87	
Iron [µg/dl] [40-120]	71.00	± 38.76	90.79	± 38.60	52.53	± 29.40	< 0.01
Transferrin [mg/dl] [200-360]	257.5	± 227.7	224.0	± 29.50	288.8	± 317.4	
Transferrin Saturation[%][15-45]	25.45	± 15.65	28.43	± 10.88	22.67	± 19.05	
Ferritin [µg/L] [30.0-400.0]	163.3	± 103.5	165.3	± 91.84	161.4	± 116.6	
Vitamin B12 [pg/mL] [197-771]	411.9	± 281.6	334.9	± 114.7	483.7	± 367.3	
Folic acid [ng/mL] [3.9-27.8]	8.08	± 5.41	8.17	± 4.21	7.99	± 6.49	
sTFR [mg/L] [0.81-1.75]	1.38	± 0.62	1.17	± 0.27	1.57	± 0.78	
Haptoglobin [mg/dl] [30-200]	131.4	± 92.2	128.2	± 54.59	134.3	± 119.2	
LDH [U/L] [<248]	392.2	± 405.2	426.2	± 468.0	360.5	± 350.3	
Bilirubin [mg/dl] [<1.00]	0.56	± 0.26	0.59	± 0.19	0.53	± 0.32	
Reticulocytes (absolute) [23-70] [x1000/µL]	79.31	± 24.56	77.43	± 13.0	81.07	± 32.27	
Ret-Hb [pg] [28-35]	34.00	± 3.67	36.19	± 1.3	31.95	± 4.01	< 0.01
CK [U/L] [<171]	393.2	± 585.1	347.1	± 364.2	436.2	± 746.6	
Troponin [ng/L] [<14]	1984	± 6090	1580	± 3845	2360	± 7755	
NT-proBNP [pg/mL] [<125]	2334	± 2529	1200	± 1589	3392	± 2823	<0.05

Values are mean ± SD. GFR= glomerular filtration rate; MCV= mean corpuscular volume; MCH= mean corpuscular haemoglobin; MCHC= mean corpuscular haemoglobin concentration; sTFR= soluble transferrin receptor; LDH= lactate dehydrogenase; Ret-Hb= reticulocytes-haemoglobin; CK= creatine kinase; NT-pro BNP= B type natriuretic peptide.

Table S9: Effects of RBC from ACS patients on left ventricular (LV) function in isolated recipient hearts with ischaemia/reperfusion

Condition	RBC Suspension	ACS without anaemia (n=14)	± SD	ACS with anaemia (n=15)	± SD	*p-value*
**Baseline**	Coronary flow [mL/min/g]	2.31	± 0.39	2.21	± 0.29	
	LV developed pressure [mmHg]	86.71	± 10.90	90.20	± 13.84	
	dP/dt_{max} [mmHg/s]	3517	± 422.3	3662	± 598.4	
	dP/dt_{min} [mmHg/s]	-2706	± 417.1	-2838	± 551.2	
**60 min**	Coronary flow [% Recovery]	2.28	± 0.31	1.73	± 0.45	< 0.001
**Recovery**	LV developed pressure [% Recovery]	29.29	± 10.86	21.67	± 8.32	< 0.05
	dP/dt_{max} [% Recovery]	1191	± 446.2	949.0	± 372.7	
	dP/dt_{min} [% Recovery]	-765.4	± 259.5	-663.4	± 231.5	
**%**	Coronary flow [% Recovery]	100.1	± 16.73	78.27	± 22.88	< 0.01
**Recovery**	LV developed pressure [% Recovery]	33.57	± 11.26	24.27	± 8.52	< 0.05
	dP/dt_{max} [% Recovery]	33.86	± 12.12	26.20	± 9.83	
	dP/dt_{min} [% Recovery]	28.50	± 9.28	23.40	± 6.61	

Data are given for coronary flow and LV function at baseline, 60 minutes after recovery and post-ischaemic recovery (%) in isolated hearts from healthy wild type mice loaded with human RBC donors of anaemic and non-anaemic ACS patients at the beginning of global myocardial ischaemia. Data are means ± SD; (n= 14 non-anaemia, n=15 anaemia). dP/dt_{max}= maximum rate of pressure increase; dP/dt_{min} = maximum rate of pressure decrease; LV = left ventricular; ACS= acute coronary syndrome.
